# Synthesis, characterization and biological evaluation of dipicolylamine sulfonamide derivatized platinum complexes as potential anticancer agents†

**DOI:** 10.1039/d1ra00842k

**Published:** 2021-05-14

**Authors:** Nadini Thushara, Taniya Darshani, Sameera R. Samarakoon, Inoka C. Perera, Frank R. Fronczek, W. M. C. Sameera, Theshini Perera

**Affiliations:** Department of Chemistry, University of Sri Jayewardenepura Sri Lanka theshi@sjp.ac.lk; Institute of Biochemistry, Molecular Biology and Biotechnology, University of Colombo Sri Lanka; Department of Zoology and Environment Sciences, University of Colombo Sri Lanka; Department of Chemistry, Louisiana State University Baton Rouge LA USA; Institute of Low Temperature Science, Hokkaido University N19-W8, Kita-ku Sapporo Hokkaido 060-0819 Japan

## Abstract

Three new Pt complexes, [PtCl_2_(N(SO_2_(2-nap))dpa)], [PtCl_2_(N(SO_2_(1-nap))dpa)] and [PtCl_2_(N(SO_2_pip)dpa)], containing a rare 8-membered ring were synthesized in good yield and high purity by utilizing the ligands N(SO_2_(2-nap))dpa, N(SO_2_(1-nap))dpa and N(SO_2_pip)dpa, which contain a dipicolylamine moiety. Structural studies of all three complexes confirmed that the ligands are bound in a bidentate mode *via* Pt–N_(pyridyl)_ bonds forming a rare 8-membered ring. The intense fluorescence displayed by the ligands is quenched upon coordination to Pt. According to time dependent density functional theory (TDDFT) calculations, the key excitations of N(SO_2_(2-nap))dpa and [PtCl_2_(N(SO_2_(1-nap))dpa)] involve the 2-nap-ligand-centered π → π* excitations. While all six compounds have shown antiproliferative activity against human breast cancer cells (MCF-7), the N(SO_2_pip)dpa and N(SO_2_(2-nap))dpa ligands and [PtCl_2_((NSO_2_pip)dpa)] complex have shown significantly high cytotoxicity, directing them to be further investigated as potential anti-cancer drug leads.

## Abstract 2

Three new Pt complexes, [PtCl_2_(N(SO_2_(2-nap))dpa)], [PtCl_2_(N(SO_2_(1-nap))dpa)] and [PtCl_2_(N(SO_2_pip)dpa)], containing a rare 8-membered ring were synthesized in good yield and high purity by utilizing ligands which contain a dipicolylamine moiety.

## Graphical abstract

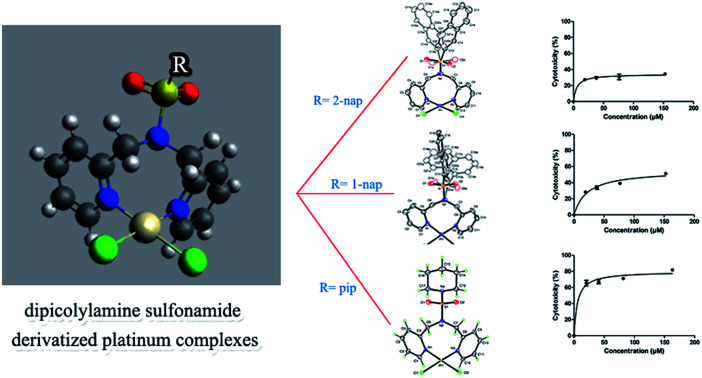

## 1. Introduction

Metals play a major role in therapeutic and diagnostic^1^ applications of cancer, prompting the exploration of novel metal organic compounds towards this end. Clinically important organometallic complexes have also been used for the synthesis of effective diagnostic agents.^2^ In fact, [Tc(CNR)_6_]^+^ is the first example of an organometallic complex to be clinically used in nuclear medicine.^3^^99m^Tc and other short lived isotopes are used as radiopharmaceuticals in disease diagnosis and treatment.^4^ Such radionuclide cationic complexes containing ^123^I nuclide^5^ and ^99m^Tc nuclide^6^ are broadly used as potential diagnostic agents while complexes bearing the ^188^Re tricarbonyl core are used as therapeutic agents.^7^ In addition to that, inorganic compounds have found use in chemotherapeutic agents such as the gold-containing antiarthritic drug auranofin and antibacterial, antiviral, antiparasitic and radiosensitizing agents.^8^

The development of modern medicinal inorganic chemistry was stimulated by the discovery of serendipitous cisplatin, the first ever anticancer drug.^9,10^ To address the drawbacks of the severe toxicity^9^ and drug resistance of cisplatin, a large number of cisplatin analogues have been synthesized and evaluated over three generations of platinum drugs (cisplatin, carboplatin and oxaliplatin).^11,12^ Pt(ii) complexes have the advantage of low coordination number, preferential binding to the more limited soft centers in proteins and nucleic acids^13^ and avoid unwanted covalent reactions with nucleotides.^14^ In fact, a key factor explaining why platinum is most useful comes from the ligand exchange kinetics, which for platinum complexes of the type *cis*-[PtX_2_(amine)_2_] (X = anionic group and amine = primary or secondary amine) are in the order of a few hours, thereby preventing rapid equilibration reactions.^15–17^ Many Pt(ii) complexes with ligands having a diene backbone have been synthesized and studied.^18^ For an example, the dansyldiene (DNSH-dienH) ligand which can act as a bidentate, tridentate or quadridentate forms a complex with platinum metal.^18^ N(SO_2_R)dpa-type tridentate ligands and their Pt(ii) compounds with chloride leaving ligands have been synthesized and studied.^19^ These N(SO_2_R)Me_*n*_dpa (R = Me, Tol; *n* = 2, 4) ligands are coordinated in a bidentate fashion in [PtCl_2_(N(SO_2_R)3,3′,5,5′-Me_4_dpa)] complexes, forming a rare eight-membered chelate ring.^19^ The recently reported dinuclear platinum-amine compounds appeared to give rise to DNA binding at two different positions, thereby enhancing the antitumor effect.^20^ Multinuclear platinum drugs that can contain two, three or four Pt centers with both *cis* and *trans* configurations have become an attractive strategy to develop potent cisplatin analogues.^15^

Breast cancer is one of the most abundant types of cancer in recent times. Among various types of chronic human diseases, certain types of cancers are widely treated by organometallic complexes.^4^ However, only a few metallopharmaceuticals are available for diagnostic and therapeutic purposes for human breast cancer.^21^

We note that sigma receptors are a specific class of membrane-bound proteins,^21^ classified as *N*-allylnormetazocine link receptors, a type of opiate receptors.^22^ These receptors, their subtypes, transmitters and enzymes are mostly found in the central nervous system, liver, kidney and can serve as attractive targets for radio-selective molecules in oncology.^22^ Sigma receptors are over expressed in vastly dividing cells such as breast, lung and prostate cancer cells^23^ and there is adequate evidence that expression of sigma receptors is down-regulated in quiescent cells.^21^ Furthermore, a few ligands labeled with ^123^I,^22^^99m^Tc,^23^^18^F^24^ have high binding affinity for sigma receptors. Caveliers *et al.* has reported on the possible use of *N*-[2-(1′-piperidinyl)ethyl]-3-^123^I-iodo-4-methoxybenzamide towards the diagnosis of patients with primary breast cancer.^21^ We ourselves have reported on the therapeutic potential of a novel sulfonamide ligand bearing a piperidinyl group and its rhenium complex, [Re(CO)_3_(N(SO_2_pip)dpa)]BF_4_ for therapy of human breast cancer, mainly because these types of compounds preferentially bind with sigma receptors.^25^

Sulfonamide groups are considered as a pharmacophore because they possess many biological activities such as antimicrobial,^26^ anticarbonic anhydrase,^27^ antihypersensitive,^28^ hypoglycemic^29^ activities as well as, most significantly, anti-cancer activity.^30^ Sulfonamide appended rhenium complexes, for example *fac*-[Re(CO)_3_(NSO_2_Rdien)]PF_6_ (R = dmb, tol) have also been proposed as model systems for radiopharmaceuticals.^31^ Symmetrically complexed radiopharmaceuticals derived from dipicolylamine, such as glucosamine-dpa^32^ have been synthesized and evaluated. Previous studies revealed that the replacement of the amine proton of N(SO_2_R)dpa by various substituents facilitates the formation of metal to nitrogen bonds within the normal range of bond lengths arising exceptional biochemical properties.^33^

**Scheme 1:**
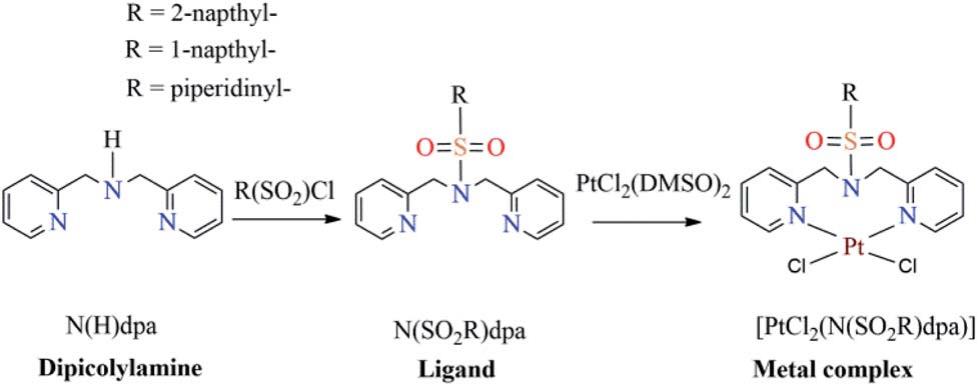
Synthetic routes for N(SO_2_R)dpa ligands and *cis*-[PtCl_2_(N(SO_2_R)dpa)].

The main objective of this study was to synthesize platinum complexes containing dipicolylamine sulfonamides and investigate their activity as anticancer agents. In this study, we specifically opted to synthesize 1-naphthalene sulfonyl, 2-naphthalene sulfonyl and piperidine-1-sulfonyl derivatized compounds based on bioactivities reported for these groups; naphthalene derivatized compounds, such as nafcillin, naftifine, tolnaftate and terbinafine, are currently being used as therapeutics^34^ while naphthalene compounds have been reported to exhibit numerous promising pharmaceutical properties, such as anticancer,^35–37^ antimicrobial,^38^ anti-inflammatory^39^ and antineurodegenerative^40,41^ activities. As noted earlier, piperidinyl derivatized compounds have been used as radiotracers towards targeting breast cancer cells.^21^ Thus, we report on the synthesis and characterization of three novel platinum complexes bearing 1-naphthalene sulfonyl, 2-naphthalene sulfonyl and piperidine-1-sulfonyl groups (Scheme 1) and evaluate their cytotoxic activity towards human breast cancer cells.

This study explores the binding of Pt(ii) towards dpa derivatized sulfonamides and presents a novel concept in terms of opening up new avenues for bioconjugation of Pt in rare eight-membered rings. It also explores the differences in coordination mode of ligands containing this scaffold towards platinum and rhenium.

## 2. Experimental

### 2.1. Materials and methods

K_2_[PtCl_4_], DMSO, di(2-picolyl)amime, piperidine-1-sulfonyl chloride, 1-naphthalene sulfonyl chloride, 2-naphthalene sulfonyl chloride, analytical grade dioxane, analytical grade methanol, chromasolv water, dichloromethane, anhydrous sodium sulphate and acetonitrile were used as received from Sigma Aldrich, USA. Human breast cancer cell line, MCF-7, was obtained from American Type Culture Collection.

### 2.2. NMR measurements

^1^H NMR spectra were recorded in DMSO-*d*_6_ using a Bruker 400 MHz spectrometer. Peak positions are relative to trimethylsilane (TMS) or solvent residual peak, with TMS as reference. All NMR data were processed with Top Spin 3.2 and Mestre-Nova software.

### 2.3. X-ray data collection and structure determination

Crystal data were collected using a Bruker Kappa APEX-(11) DUO diffractometer. Data were collected at low temperature and data reduction was done on Bruker SAINT and includes absorption by multi scan method, using SHELXS97. Molecular graphics were drawn using ORTEP-3 for windows.

### 2.4. UV-visible spectroscopy

Electronic spectra for ligands and metal complexes were obtained within the spectral range of 200–800 nm using Spectro VIS auto version 3.16, UV-2602 spectrometer. Methanol was used to obtain the spectra with baseline correction. Spectral data were processed with UV WIN software.

### 2.5. FTIR analysis

ATR spectra for ligands and metal complexes were obtained within the spectral range of 4000–600 cm^−1^ using Thermo Scientific NICOLET iS10 spectrometer. Spectral data were processed with OMNIC software.

### 2.6. Fluorometric analysis

Emission spectra for ligands and metal complexes were obtained in methanol on a Thermo Scientific Lumina spectrophotometer. A 150 W xenon lamp was used as the excitation source. Spectral data were processed with Luminous software.

### 2.7. Melting point determination

Melting points were manually determined in open capillaries.

### 2.8. Synthesis

In order to synthesize metal complexes, [PtCl_2_(DMSO)_2_] precursor was prepared as the starting material using K_2_[PtCl_4_] according to a known procedure.^42^

#### 2.8.1. N(SO_2_(2-nap))dpa ligand

A solution of 2-naphthalene sulfonyl chloride (2.5 mmol, 0.572 g) was added to a solution of N(H)dpa (5 mmol, 0.92 ml) according to a known procedure to obtain N(SO_2_(2-nap))dpa ligand.^43^ Brownish yellow colour, plate like crystals suitable for X-ray diffraction were obtained (0.662 g, 68%). ^1^H NMR, UV-vis and FTIR data were matched with previously reported data. Melting point: 80.5 °C.

#### 2.8.2. [PtCl_2_(N(SO_2_(2-nap))dpa)] complex

A solution of N(SO_2_(2-nap))dpa (0.1 mmol, 0.039 g) in 10 ml of ethanol was added to a solution of [PtCl_2_(DMSO)_2_] (0.1 mmol, 0.042 g) in 10 ml of ethanol at room temperature. The reaction mixture was stirred at 50 °C for 12 hours. Then the resultant precipitate was filtered using a filter paper. The precipitate was taken to dryness. White color powder was obtained (0.059 g, 90%). UV-vis (MeOH) [*λ*_max_ (nm) (*ε* M^−1^ cm^−1^)]: 228, 269; FT-IR (ATR) (cm^−1^): 3052 (*ν*(C–H)), 1336 (*ν*(C–N)), 890 (*ν*(S–N)). ^1^H NMR signals (ppm) in DMSO-*d*_6_ are 9.27 (d, 2H, H6/6′), 8.74 (s, 1H), 8.24 (t, 1H), 8.12 (d, 1H), 8.03 (d, 1H), 7.96 (t, 2H, H4/4′), 7.79–7.75 (m, 2H), 7.74 (d, 2H, H3/3′), 7.52 (t, 2H, H5/5′), 6.06 (d, *endo*-H), 5.33 (d, *exo*-H). Crystals suitable for single crystal X-ray diffraction were grown by mixing two solutions, each containing 12.5 mM ligand and platinum precursor in acetonitrile. Melting point > 360 °C.

#### 2.8.3. N(SO_2_(1-nap))dpa ligand

A solution of 1-naphthalene sulfonyl chloride (2.5 mmol, 0.572 g) was added to a solution of N(H)dpa (5 mmol, 0.92 ml) according to a known procedure to obtain N(SO_2_(2-nap))dpa ligand as a brown coloured oil (0.681 g, 70%).^43^^1^H NMR, UV-vis and FTIR data matched with previously reported data.

#### 2.8.4. [PtCl_2_(N(SO_2_(1-nap))dpa)] complex

A solution of N(SO_2_(1-nap))dpa (0.1 mmol, 0.039 g) in 10 ml of ethanol was added to a solution of [PtCl_2_(DMSO)_2_] (0.1 mmol, 0.042 g) in 10 ml of ethanol at room temperature. The reaction mixture was stirred at 50 °C for 12 hours. Then the resultant precipitate was filtered using a filter paper and the resulting precipitate was taken to dryness. Yellow-white color powder was obtained (0.050 g, 77%). UV-vis (MeOH) [*λ*_max_ (nm) (*ε* M^−1^ cm^−1^)]: 208, 222, 275; FT-IR (ATR) (cm^−1^): 3071 (*ν*(C–H)), 1326 (*ν*(C–N)), 899 (*ν*(S–N)). ^1^H NMR signals (ppm) in DMSO-*d*_6_ are 9.29 (d, 2H, H6/6′), 8.42 (d, 1H), 8.34 (d, 1H), 8.29 (d, 1H), 8.13 (d, 1H), 7.89 (t, 2H, H4/4′), 7.76 (t, 1H), 7.67 (t, 1H), 7.60 (t, 1H), 7.55–7.49 (m, 4H, H5/5′ and H3/3′), 6.42 (d, *endo*-H), 5.33 (d, *exo*-H). Melting point > 360 °C. Crystals suitable for single crystal X-ray diffraction were grown as described in Section 2.8.2.

#### 2.8.5. N(SO_2_pip)dpa ligand

A solution of piperidine-1-sulfonyl chloride (2.5 mmol, 0.459 g) was added to a solution of N(H)dpa (5 mmol, 0.927 ml) according to a known procedure to obtain N(SO_2_pip)dpa ligand.^25^ Grey color, plate like crystals were obtained (0.814 g, 94%) and ^1^H NMR, UV-vis and FTIR data were matched with previously reported data.^25^ Melting point: 77.5 °C.

#### 2.8.6. [PtCl_2_(N(SO_2_pip)dpa)] complex

A solution of N(SO_2_pip)dpa (0.1 mmol, 0.035 g) in 10 ml of ethanol was added to a solution of [PtCl_2_(DMSO)_2_] (0.1 mmol, 0.042 g) in 10 ml of ethanol at room temperature after which the reaction mixture was stirred at 50 °C for 8 hours. The resultant precipitate was filtered using a filter paper and dried to yield white color, powder (0.098 g, 60%). UV-vis (MeOH) [*λ*_max_ (nm) (*ε* M^−1^ cm^−1^)]: 203, 243, 267; FT-IR (ATR) (cm^−1^): 2940 (*ν*(C–H)), 1426 (*ν*(C–N)), 923 (*ν*(S–N)). ^1^H NMR signals (ppm) in DMSO-*d*_6_ are 3.12 (d, 2H, H_a_), 1.50 (t, 3H, H_b_), 9.25 (d, 2H, H6/6′), 7.92 (t, 2H, H4/4′), 7.57 (d, 2H, H3/3′), 7.49 (t, 2H, H5/5′), 6.18 (d, *endo*-H), 5.14 (d, *exo*-H). Melting point > 360 °C. Crystals suitable for single crystal X-ray diffraction were grown as detailed in Section 2.8.2.

### 2.9. Computational methods

Geometry optimizations were performed using density functional theory (DFT) as implemented in the Gaussian 16 program.^44^ The PBE1PBE^45^ density functional, including the Grimme's dispersion^46^ and the Becke–Johnson damping, was employed for ground state calculations. The SDD^47,48^ basis set and associated effective core potentials were used for Pt, and the det2-TZVP^49,50^ basis sets were applied for the other atoms. The polarizable continuum model (PCM)^51–53^ was used as the implicit solvation model, where methanol (*ε* = 32.613) was the solvent. Excited-state calculations were performed using the time dependent density functional theory (TDDFT), where the PBE1PBE functional with the Grimme's dispersion and the Becke–Johnson damping, PCM solvation, and the basis sets described above were employed. The nonequilibrium PCM was applied for calculating the singlet vertical excitations, while the equilibrium PCM was used for optimizing the excited states. The “UltraFine” integration grid was applied for TDDFT calculations, where the two-electron integral accuracy parameter was set to 12. All geometry optimizations were full with no restrictions. Vibrational frequency calculations (at 298.15 K and 1 atm) confirmed that the optimized ground structures were minima (*i.e.* no imaginary frequencies).

### 2.10. Biological studies

#### 2.10.1. Anticancer activity

The ligands and corresponding metal complexes were investigated for their cytotoxicity against MCF-7 (breast cancer) cells and MCF-10A (normal human breast) cells. Cells were cultured in 96-well culture plates and exposed to 12.5 μg ml^−1^, 25 μg ml^−1^, 50 μg ml^−1^ and 100 μg ml^−1^ concentrations of ligands and complexes for 24 hours and cytotoxicity was assessed by sulforhodamine B assay. All exposures were carried out in triplicate. Briefly, the cell supernatant was completely removed and washed with phosphate buffer solution. Trichloroacetic acid (50%, 25 μl) was added on top of fetal bovine serum-free fresh medium (200 μl) to make final concentration of 10% trichloroacetic acid and was incubated at 4 °C for one hour prior to the SRB assay. The plates were then washed with five washing cycles with water and dried completely. An aliquot of 100 μl of 0.4% sulforhodamine B dissolved in 1% trichloroacetic acid, was added to each well, and was allowed to stain for 15 minutes. The plates were again washed with five washing cycles to remove unbound dye using 1% (vol/vol) acetic acid after removing the stain. The protein bound dye was solubilized with trisbase (10 mM, pH 7.5, 200 μl), after air drying. The plates were then shaken for 60 minutes to homogenize the dye solution. The absorbance was then measured at 540 nm using Synergy HTBioTek microplate reader. The percentage viability was calculated by the equation given below.1
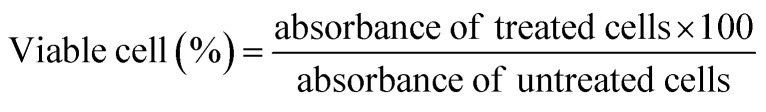


## 3. Results and discussion

### 3.1. X-ray crystallography

**Fig. 1:**
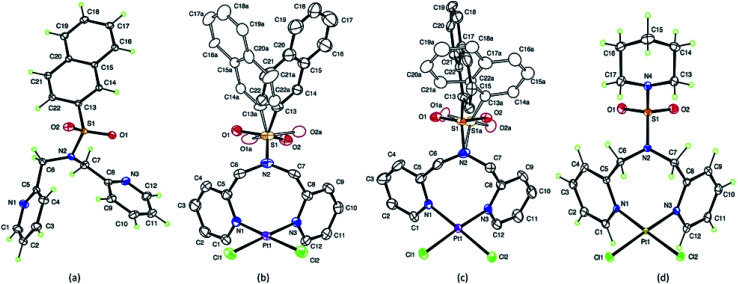
ORTEPs of N(SO_2_(2-nap))dpa (a), [PtCl_2_(N(SO_2_(2-nap))dpa)] (b), [PtCl_2_(N(SO_2_(1-nap))dpa)] (c) and [PtCl_2_(N(SO_2_pip)dpa)] (d). Thermal ellipsoids are drawn with 50% probability with naphthyl disorder shown in (b) and (c).

Crystal data and details of the structural refinement for N(SO_2_(2-nap))dpa, [PtCl_2_(NSO_2_(2-nap)dpa)], [PtCl_2_(NSO_2_(1-nap)dpa)] and [PtCl_2_((NSO_2_pip)dpa)] are summarized in Table 1. Crystallographic data are deposited with the Cambridge Crystallographic Data Centre under deposition numbers CCDC 1867219, 1867220, 1867221 and 1867222. Crystal data and details of the structural refinement for N(SO_2_pip)dpa^25^ were utilized for comparison purposes. The ORTEP plots of the compounds; N(SO_2_(2-nap))dpa, [PtCl_2_(N(SO_2_(2-nap))dpa)], [PtCl_2_(N(SO_2_(1-nap))dpa)] and [PtCl_2_(N(SO_2_pip)dpa)] are given in Fig. 1. Key structural parameters of the X-ray structures and fully optimized ground state structures are summarized in Table 2. In general, calculated structures are in agreement with the X-ray structures.

**Table 1:** Crystal data and structure refinement for N(SO_2_(2-nap))dpa, [PtCl_2_(NSO_2_(2-nap))dpa], [PtCl_2_(NSO_2_(1-nap))dpa] and [PtCl_2_(NSO_2_pipdpa)]

Crystal data	N(SO_2_(2-nap))dpa	[PtCl_2_(N(SO_2_(2-nap))dpa)]	[PtCl_2_(NSO_2_(1-nap)dpa)]	[PtCl_2_((NSO_2_pip)dpa)]
Empirical formula	C_22_H_19_N_3_O_2_S	C_22_H_19_Cl_2_N_3_O_2_PtS	C_22_H_19_Cl_2_N_3_O_2_PtS	C_17_H_22_Cl_2_N_4_O_2_PtS
*M*_r_	389.46	655.45	655.45	612.43
Crystal description	Lath, colorless	Needle, colorless	Lath, colorless	Needle, yellow
Crystal system	Monoclinic	Monoclinic	Monoclinic	Monoclinic
Space group	*P*2_1_/*c*	*C*2/*c*	*C*2/*c*	*I*2/*a*
Crystal size (mm)	0.48 × 0.31 × 0.07	0.26 × 0.06 × 0.02	0.30 × 0.10 × 0.02	0.16 × 0.10 × 0.04
Temperature (K)	90	100	100	90

**Unit cell dimensions**				
*a* (Å)	20.8714 (8)	25.9376 (10)	26.7076 (7)	17.5316 (5)
*b* (Å)	5.6704 (2)	9.6371 (3)	8.7572 (2)	9.5135 (2)
*c* (Å)	17.1989 (6)	17.6498 (6)	18.7201 (4)	24.2020 (9)
*β* (deg)	114.2072 (18)	97.188 (4)	91.356 (1)	91.872 (2)
*V* (Å^3^)	1856.49 (12)	4377.1 (3)	4377.10 (18)	4034.4 (2)
*Z*	4	8	8	8
*R*[*F*^2^ > 2*σ*(*F*^2^)]	0.035	0.030	0.037	0.040
w*R*(*F*^2^)	0.102	0.070	0.080	0.077
Data/parameters	9447/253	6705/371	8404/398	11 296/244
Radiation type	Mo Kα	Mo Kα	Mo Kα	Mo Kα
Radiation wavelength (Å)	0.71073	0.71073	0.71073	0.71073
Abs coeff/*μ* (mm^−1^)	0.20	6.78	6.78	7.35
2*θ* max (deg)	74.2	61.2	66.6	77.2

**Table 2:** Selected bond distances (Å) and bond angles (°) of [PtCl_2_(N(SO_2_(2-nap))dpa)], [PtCl_2_(N(SO_2_(1-nap))dpa)] and [PtCl_2_(N(SO_2_pip)dpa)]

	[PtCl_2_(N(SO_2_(2-nap))dpa)]		[PtCl_2_(N(SO_2_(1-nap))dpa)]		[PtCl_2_(N(SO_2_pip)dpa)]	
	X-ray	Calculated	X-ray	Calculated	X-ray	Calculated
Pt1–N1	2.028 (3)	2.05	2.029 (3)	2.05	2.033 (2)	2.05
Pt1–N3	2.030 (3)	2.06	2.027 (4)	2.06	2.017 (3)	2.06
Pt1–Cl1	2.2930 (9)	2.33	2.2947 (10)	2.33	2.2996 (8)	2.33
Pt1–Cl2	2.2980 (9)	2.34	2.2955 (11)	2.34	2.3048 (7)	2.34
S1–O1	1.532 (5)	1.43	1.417 (11)	1.43	1.432 (3)	1.43
S1–O2	1.350 (5)	1.43	1.457 (7)	1.43	1.428 (3)	1.43
S1–N2	1.627 (3)	1.63	1.575 (5)	1.63	1.638 (3)	1.64
S1–N4	—	—	—	—	1.632 (3)	1.63
S1–O1A	1.340 (7)	—	1.423 (15)	—	—	—
S1–O2A	1.601 (7)	—	1.441 (9)	—	—	—
N3–Pt1–N1	90.05 (11)	89.67	93.98 (14)	88.91	91.06 (10)	88.16
N3–Pt1–Cl1	177.34 (9)	178.23	174.89 (10)	177.88	176.44 (7)	177.23
N1–Pt1–Cl1	89.03 (8)	88.98	86.79 (10)	89.29	88.52 (8)	89.36
N3–Pt1–Cl2	89.16 (8)	89.01	87.44 (11)	89.19	88.52 (7)	89.80
N1–Pt1–Cl2	178.95 (8)	178.14	178.56 (10)	177.71	179.05 (7)	177.39
Cl1–Pt1–Cl2	91.79 (4)	92.30	91.77 (4)	92.58	91.96 (3)	92.64
C7–N2–C6	121.8 (3)	121.1	120.6 (4)	120.7	120.1 (3)	120.2
C7–N2–S1	118.4 (3)	117.5	124.9 (4)	118.8	118.7 (2)	118.9
C6–N2–S1	118.9 (2)	120.4	111.5 (3)	118.9	118.6 (2)	119.2
O2–S1–O1	120.0 (3)	119.7	121.0 (5)	119.2	121.56 (16)	120.6
O2–S1–N4	—	—	—	—	106.23 (15)	106.7
O1–S1–N4	—	—	—	—	106.21 (15)	106.7
O2–S1–N2	109.1 (2)	108.4	102.5 (3)	107.3	105.64 (15)	106.4
O1–S1–N2	104.6 (2)	106.5	110.9 (5)	107.3	105.54 (15)	106.1
N4–S1–N2	—	—	—	—	111.69 (15)	110.2
O1A–S1A–N2	110.8 (3)	—	101.3 (7)	—	—	—
O2A–S1A–N2	105.1 (3)	—	111.5 (4)	—	—	—

The S–N bond length (1.6277 (6) Å) of the N(SO_2_(2-nap))dpa ligand is comparable with the S–N bond lengths of 1.6194 (11) Å for N(SO_2_pip)dpa^25^ and 1.602 (9) Å for *N*-methyltoluene-*p*-sulfonamide and 1.641 (2) Å for *N*,*N*-dimethyltoluene-*p*-sulfonamide.^54^ However, that has not been depicted by the shortening of S–N bond length in N(SO_2_(2-nap))dpa as that of the most common cases.^55–57^ In the ligand, the bond distances between methylene carbon and pyridyl nitrogen (1.4752(9) and 1.471 (9) Å), have not deviated from that of the normal range^56^ for C–N sp^3^ bonds. This C6–N2 bond distance (pyridyl N and methylene carbon) of bidentate [PtCl_2_(N(SO_2_pip)dpa)] (1.477 (4) Å) complex is not significantly different to that of tridentate [Re(CO)_3_(N(SO_2_pip)dpa)]^+^ (1.4615 (16) Å) complex.^25^

In [PtCl_2_(N(SO_2_(1-nap))dpa)], Pt1–N3 and Pt1–N1 (2.029 Å, 2.027 Å) bond distances are similar to Pt–N bond lengths in compounds of the type [PtL_2_X_2_] where L = aniline and X = halides and nitrites,^58^ illustrating that the interaction between the new ligand and Pt is strong. The Cl1–Pt1–Cl2 angle (91.79° (4)) is the highest angle around Pt atom because of the high repulsion of lone pairs of the two chlorine atoms. The obtained N1–Pt1–N3 bond angle is 90.05° (11) and there is no significant change in bond angles of atoms in pyridyl ring except *endo* nitrogen atom upon complex formation. The S–O bond distances of these tridentate ligands are somewhat similar to that of S=O in bidentate (N(SO_2_L)dpa) ligands.^25^ Further, Pt–Cl bonds are in the range of normal bond lengths^55^ and provide evidence that there is no change of Pt–Cl bond after complexation. The bond angle of Cl1–Pt1–Cl2 (91.77° (4)) of the [PtCl_2_(N(SO_2_(1-nap))dpa)] complex is fairly similar to that of [PtCl_2_(N(SO_2_(2-nap))dpa)] complex. The bond length of S1–C13A of the [PtCl_2_(N(SO_2_(1-nap))dpa)] complex is 1.770 Å and is higher than that of [PtCl_2_(N(SO_2_(2-nap))dpa)] complex. These [PtCl_2_(N(SO_2_(2-nap))dpa)] and [PtCl_2_(N(SO_2_(1-nap))dpa)] complexes show disorder in which sulfur atom has bonded in two different angles to the central nitrogen atom of dipicolylamine group. The two conformers are present in unequal amounts of 56 : 44.

For the [PtCl_2_(N(SO_2_pip)dpa)] complex, the structural data revealed that the S–O bond lengths (Table 2) are similar to that of S=O in SO_2_ (1.43 Å).^54^ Since the O–S–O angle is as wide as to 121.56 (16) Å and the other angles around sulfur atom are slightly smaller in the range of 106.10–108.17 Å (Table 3) than the ideal tetrahedral angle (109.5°), the sulfonyl moiety represents a distorted tetrahedral arrangement around the S atom.

**Table 3:** Comparison of ^1^H NMR shifts (ppm) of dipicolylamine units of ligands and complexes in DMSO-*d*_6_

Compound	H6/6′	H5/5′	H4/4′	H3/3′	–CH_2_
N(SO_2_(2-nap))dpa	8.31 (d)	7.15 (t)	7.63 (t)	7.28 (d)	4.60 (s)
[PtCl_2_(N(SO_2_(2-nap))dpa)]	9.27 (d)	7.52 (t)	7.96 (t)	7.74 (d)	6.06 (d), 5.33 (d)
N(SO_2_(1-nap))dpa	8.35 (d)	7.18 (t)	7.58 (t)	7.13 (d)	4.72 (s)
[PtCl_2_(N(SO_2_(1-nap))dpa)]	9.29 (d)	7.49 (t)	7.89 (t)	7.55 (d)	6.42 (d), 5.32 (d)
[PtCl_2_(N(SO_2_pip)dpa)]	9.25 (d)	7.49 (t)	7.92 (t)	7.57 (d)	6.18 (d), 5.14 (d)

### 3.2. ^1^H NMR analysis

**Fig. 2:**
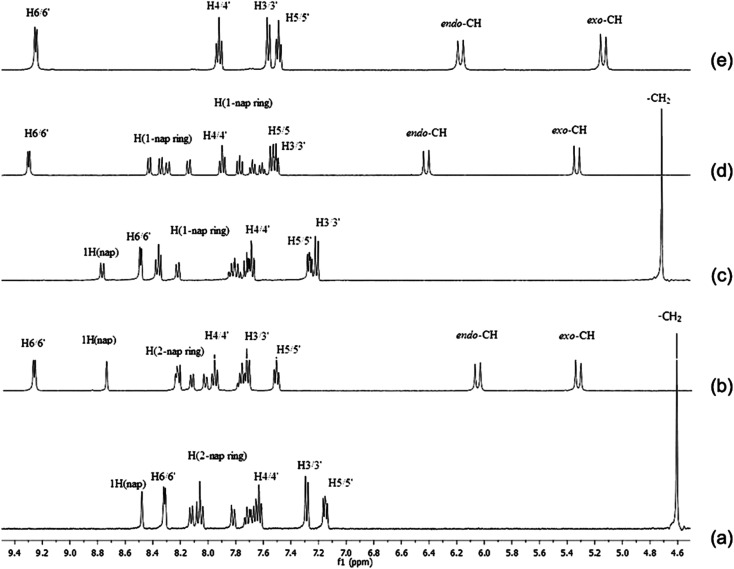
The ^1^H NMR spectra of (a) N(SO_2_(2-nap))dpa, (b) [PtCl_2_(N(SO_2_(2-nap))dpa)], (c) N(SO_2_(1-nap))dpa, (d) [PtCl_2_(N(SO_2_(1-nap))dpa)] and (e) [PtCl_2_(N(SO_2_pip)dpa)] in DMSO-*d*_6_ at 298 K.

The newly synthesized platinum complexes, [PtCl_2_(N(SO_2_(2-nap))dpa)], [PtCl_2_(N(SO_2_(1-nap))dpa)] and [PtCl_2_(N(SO_2_pip)dpa)] were characterized by ^1^H NMR spectroscopy in DMSO-*d*_6_ at 298 K. All the peaks were assigned related to the structure of metal complex, based on the chemical shifts, splitting patterns as well as the integration of corresponding peaks. Peaks related to the residual solvents were also identified.^59^^1^H NMR data for the ligands utilized in this study have been previously reported.^25,43^ Therefore, the signals related to the platinum complexes were assigned accordingly. ^1^H NMR spectra for all compounds are depicted in Fig. 2. Comparison of ^1^H NMR shifts (ppm) of dipicolylamine units of ligands and complexes in DMSO-*d*_6_ is reported in Table 3. In the ^1^H NMR spectrum of N(SO_2_(2-nap))dpa, the aromatic region (7.66–8.13 ppm) is crowded with signals due to magnetically inequivalent protons present in 2-naphthalene group of the ligand. The signal at 7.63 ppm corresponds to the H4/4′ proton that is in the *para* position of the pyridyl nitrogen. Then in the range of 7.66 ppm to 8.13 ppm, 2-naphthalene ring protons can be identified according to the previously reported data for the naphthalene ring.^60^ Within that region, the proton attached to C14 (1H nap in Fig. 2) at 8.47 ppm can be clearly identified due to the fact that it is a singlet and is the most deshielded proton in the naphthalene ring. Two protons in each methylene group are sterically similar. Hence, their apparent magnetic equivalence is depicted in ^1^H NMR spectra by a singlet found at 4.60 ppm.

In the [PtCl_2_(N(SO_2_(2-nap))dpa)] complex, the peak attributed to methylene protons has split into two doublets (6.06 ppm and 5.33 ppm) due to magnetic inequivalence and are shifted downfield. Upon coordinating to Pt, the H6/6′ doublet has moved downfield depicting coordination of N1 and N3 to Pt. Furthermore, the proton (attached to C14) of [PtCl_2_(N(SO_2_(2-nap))dpa)] appears as a singlet at 8.74 ppm.

In a ^1^H NMR spectrum of the N(SO_2_(1-nap))dpa ligand, 1-naphthalene ring protons can be identified in the range of 7.65 ppm to 8.21 ppm. Similar to N(SO_2_(2-nap))dpa, the proton of 1-naphthalene ring (attached to C14, Fig. 1) at 8.58 ppm can be clearly identified. Two protons of methylene group are sterically similar. Therefore a singlet for those methylene protons was found at 4.72 ppm (Fig. 2). Methylene protons of [PtCl_2_(N(SO_2_(1-nap))dpa)] complex has split into two doublets (6.42 ppm and 5.32 ppm) due to magnetic inequivalence and are shifted downfield. Upon coordinating to Pt, the H6/6′ doublet and 1H singlet of [PtCl_2_(N(SO_2_(1-nap))dpa)] have moved downfield (9.29 ppm and 8.74 ppm, respectively).

In the [PtCl_2_(N(SO_2_pip)dpa)] complex, a higher downfield shift (0.77 ppm) is observed *vs.* 0.39 ppm for the rhenium tricarbonyl complex bearing the same ligand^25^ showing the greater inductive of Pt *vs.* Re. A similar pattern was observed for Pt *vs.* Re complexes carrying the naphthyl derivatives.^43^

For all the signals in the ligand upon coordination to Pt, significant downfield chemical shifts are observed (Table 3). These chemical shifts provide a strong evidence for the formation of metal complex. Downfield chemical shifts are due to inductive effect resulting from the direct Pt–N bonds which are formed between Pt metal and N atoms of the pyridyl ring.

### 3.3. FTIR analysis

As evident from literature, the short absorption band at 3052 cm^−1^ represents the asymmetric stretching vibrations of C–H bonds in aromatic ring whereas the short absorption peaks around 2900 cm^−1^ are attributed to the C–H symmetric stretching vibrations in aliphatic systems in N(SO_2_(2-nap))dpa ligand and its [PtCl_2_(N(SO_2_(2-nap))dpa)] complex. The spectra exhibited the decrease of intensities of the bonds at 1426 cm^−1^ to 1608 cm^−1^ which may be attributed to the C=C groups in the naphthalene ring. Consequently, the stretching vibrational peak due to C=N, in the pyridyl ring may be present in the range between 1426 cm^−1^ to 1590 cm^−1^. The absorption peaks at 928 cm^−1^ (N(SO_2_(2-nap))dpa), 890 cm^−1^ ([PtCl_2_(N(SO_2_(2-nap))dpa)]), 920 cm^−1^ (N(SO_2_(1-nap))dpa), 899 cm^−1^ ([PtCl_2_(N(SO_2_(1-nap))dpa)]) and 923 cm^−1^ ([PtCl_2_(N(SO_2_pip)dpa)]) are due to S–N stretching vibrational modes for sulfonamide groups. Most of the ligand peaks also appear in the spectra of new complexes. The strong peak at 1334 cm^−1^ and 1332 cm^−1^ were attributed to S–C stretching vibrations of the bond between S atom of sulfonyl group and the C atom of naphthalene group of N(SO_2_(2-nap))dpa and [PtCl_2_(N(SO_2_(2-nap))dpa)], respectively.

Similar to N(SO_2_(2-nap))dpa, the short absorption band at 3071 cm^−1^ represents the asymmetric stretching vibrations of C–H bonds in aromatic ring of 1-naphthalene. C=C groups in the naphthalene ring exhibits peaks at 1590 cm^−1^ and 1473 cm^−1^. The stretching vibrational peak due to newly formed S–C may be present at 1276 cm^−1^. Most of the other peaks are similar to the previously discussed ligand, N(SO_2_(2-nap))dpa. According to the results obtained, most of the ligand peaks also appear in the spectrum of new [PtCl_2_(N(SO_2_(1-nap))dpa)] complex. The peaks at 1457 cm^−1^ and 1598 cm^−1^ are due to C=C stretching vibrations of naphthalene ring. The peaks at 1276 cm^−1^ and 1132 cm^−1^ were attributed to S–C stretching vibrations of N(SO_2_(1-nap))dpa and [PtCl_2_(N(SO_2_(1-nap))dpa)], respectively. The obtained sharp peaks at 1157 cm^−1^ and 1134 cm^−1^ of metal precursor due to the S–Pt bond were not observed in the spectrum of [PtCl_2_(N(SO_2_pip)dpa)] complex providing evidence that N(SO_2_pip)dpa ligand and metal precursor have completely reacted to give the product of [PtCl_2_(N(SO_2_pip)dpa)].

### 3.4. UV-visible analysis

**Fig. 3:**
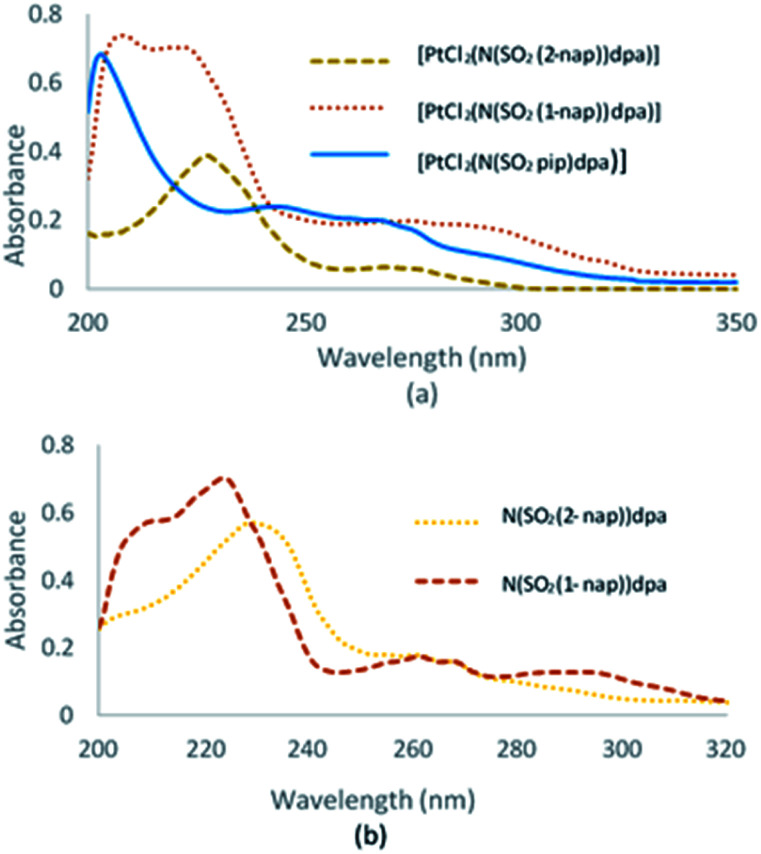
UV-visible spectra of Pt complexes (a) and ligands (b).

UV-visible spectra of N(SO_2_(2-nap))dpa, [PtCl_2_(N(SO_2_(2-nap))dpa)], N(SO_2_(1-nap))dpa, [PtCl_2_(N(SO_2_(1-nap))dpa)] and [PtCl_2_(N(SO_2_pip)dpa)] in methanol are given in Fig. 3.

**Fig. 4:**
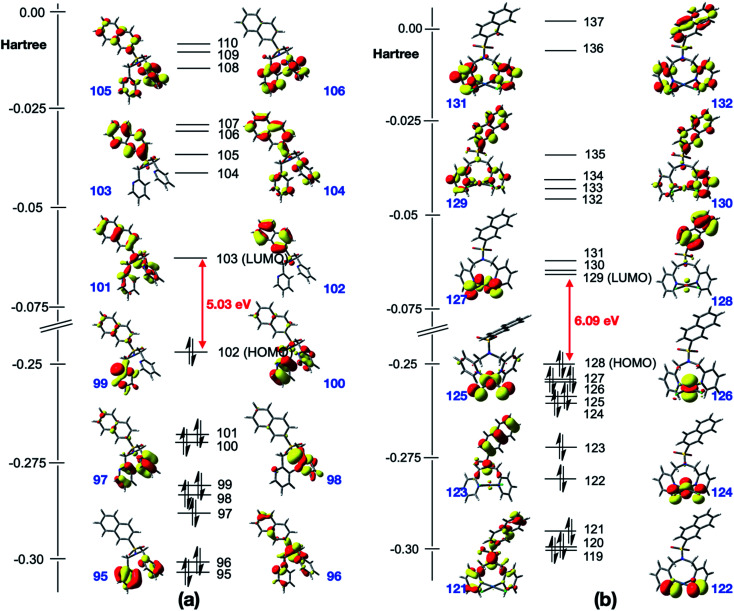
Kohn–Sham frontier molecular orbitals of the optimized structures of (a) N(SO_2_(2-nap))dpa and (b) [PtCl_2_(N(SO_2_(2-nap))dpa)].

UV-visible data for N(SO_2_pip)dpa was previously reported.^25^ Kohn–Sham frontier molecular orbitals of the ground state optimized structures of N(SO_2_(2-nap))dpa and [PtCl_2_(N(SO_2_(2-nap))dpa)] complex are shown in Fig. 4 (see Fig. S2† for the Kohn–Sham frontier molecular orbitals of [PtCl_2_(N(SO_2_(1-nap))dpa)] and [PtCl_2_(N(SO_2_pip)dpa)] complexes). In both systems, the HOMO and LUMO are delocalized on the nap unit. Computed HOMO–LUMO gap of N(SO_2_(2-nap))dpa is 5.03 eV, and [PtCl_2_(N(SO_2_(2-nap))dpa)] has a slightly higher HOMO–LUMO gap (6.09 eV). For the N(SO_2_(2-nap))dpa system, calculated natural transition orbitals (NTO) indicated four types of π → π* excitations; (i) π (nap) → π* (nap) at 289 nm (*f* = 0.06), (ii) π (dpa) → π* (nap) at 244 nm (*f* = 0.24) and 243 (*f* = 0.30), (iii) π (dpa) → π* (dpa) at 223 nm (*f* = 0.14), (iv) π (nap) → π* (dpa) at 211 nm (*f* = 0.12). This is qualitatively in agreement with the experimental spectrum and previous studies.^43^ In the case of [PtCl_2_(N(SO_2_(2-nap))dpa)] complex, NTOs indicated that the key excitations at 291 nm (*f* = 0.05) and 279 nm (*f* = 0.05) have metal to dpa ligand charge transfer character, while the excitation at 277 nm (*f* = 0.0.03) has the 2-nap-ligand-centered π → π* character, which is qualitatively in agreement with the experimental data (612 nm emission).

### 3.5. Fluorometric analysis

Fluorescence spectra were obtained for all six compounds (Table S3†) in methanol. The concentration of the test samples were approximately 0.01 mol dm^−3^ for the analysis. Fluorescence spectrum for N(SO_2_pip)dpa was previously reported.^25^ The compounds were excited in the UV range and emission spectra of Pt complexes were obtained with *λ*_max_ = 275 nm (Fig. S1†). In addition to that, a small peak was observed at 757 nm for the three complexes (Table S3†).

**Fig. 5:**
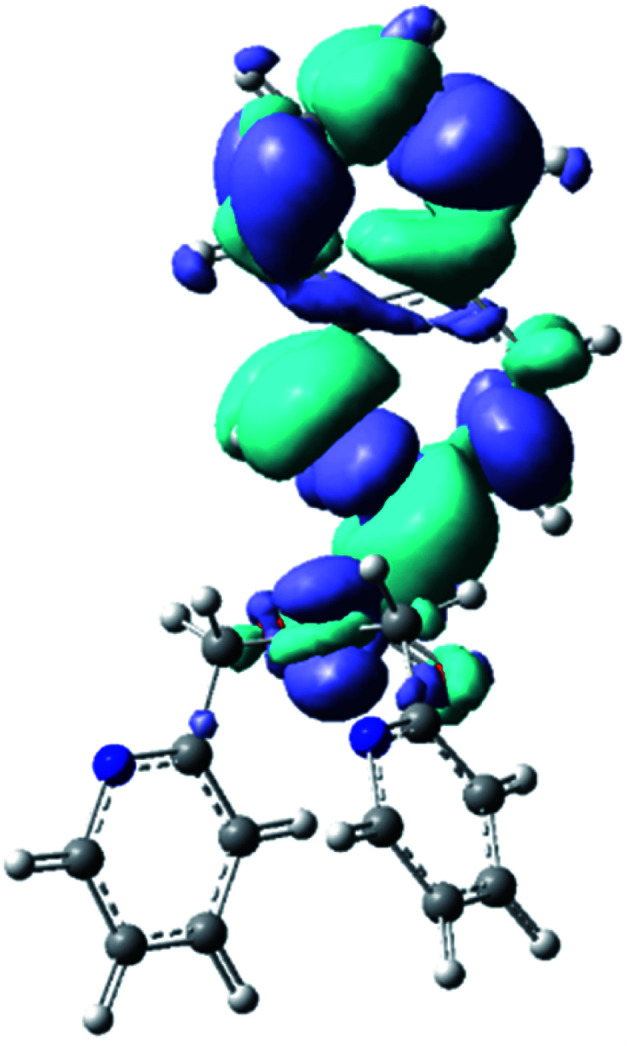
Calculated electron density difference between the singlet excited state minima and its ground state of N(SO_2_(2-nap))dpa system.

We have calculated the lowest excited singlet state minima for N(SO_2_(2-nap))dpa system, showing the emission at 354 nm. This is in agreement with the experimental value (343 nm). Computed electron density difference between the singlet excited state minima and its ground state is shown in Fig. 5, which can be assigned as the 2-nap ligand-centered emission (*i.e.*^1^LC).

### 3.6. Bio assays

#### 3.6.1. Antiproliferative activity

**Fig. 6:**
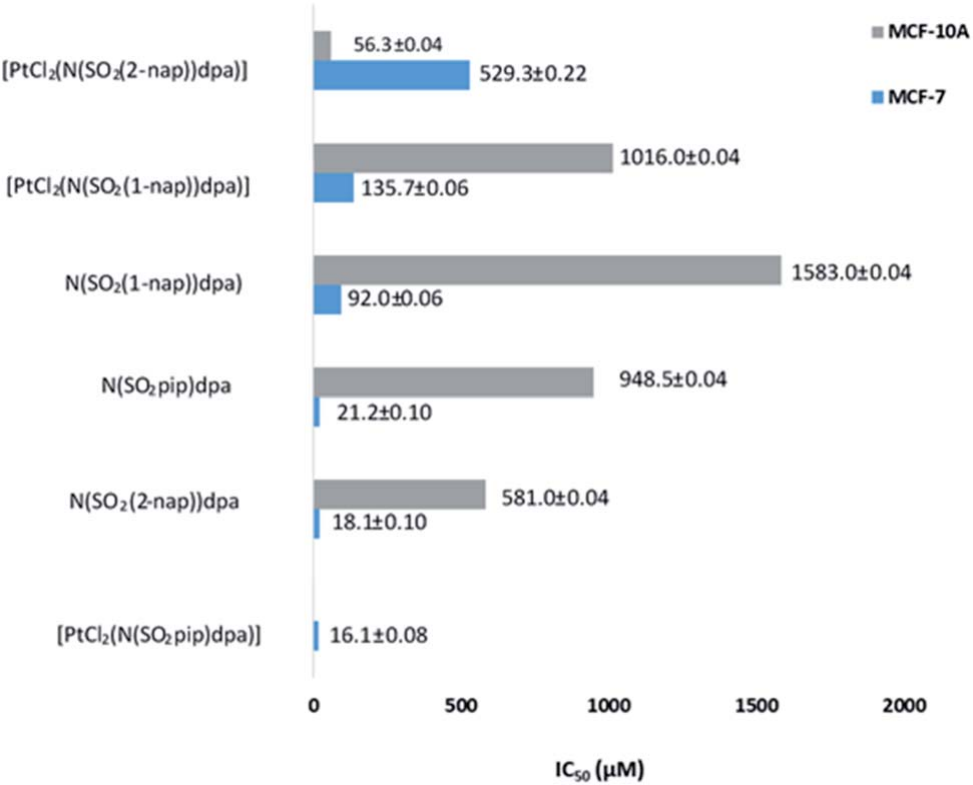
Comparison of IC_50_ values of the test compounds against human normal breast cells (MCF-10A) and human breast cancer cells (MCF-7).

Three ligands and three novel complexes were assayed for their antiproliferative activity in human breast cancer cells (MCF-7) and normal human breast cells (MCF-10A). In this study, the cell lines were exposed to synthesized compounds in a concentration gradient up to 100 mg ml^−1^ and the half maximal inhibitory concentration (IC_50_) was determined for each compound. Cytotoxicity was measured with sulforhodamine B assay. The results are provided in Fig. 6.

Excitingly, all ligands and complexes show dose dependent antiproliferative activity against the MCF-7 human breast cancer cell line where N(SO_2_(1-nap))dpa, N(SO_2_(2-nap))dpa and N(SO_2_pip)dpa show IC_50_ values of 92.0 ± 0.06 μM, 18.1 ± 0.10 μM and 21.2 ± 0.10 μM, respectively. High level of cytotoxicity demonstrated by the ligands against MCF-7 breast cancer cells shows their potential to be effective drug leads. Furthermore, their high potency for the usage as drug leads is strengthened by their lower toxicity towards MCF-10A normal human breast cells (N(SO_2_(1-nap))dpa) (IC_50_ 1583.0 ± 0.04 μM), N(SO_2_(2-nap))dpa (IC_50_ 581.0 ± 0.04 μM) and N(SO_2_pip)dpa (IC_50_ 948.5 ± 0.04 μM).

Interestingly, platinum complexation changes the dynamics of the ligands' behavior against the cancer cells as well as the normal cells. [PtCl_2_(N(SO_2_(1-nap))dpa)] compared to its ligand has low cytotoxicity against the MCF-7 but higher cytotoxicity against MCF-10A. Furthermore, N(SO_2_(1-nap))dpa shows a biphasic cytotoxicity curve suggesting the involvement of multiple pathways in eliciting cytotoxic activity (Fig. S3†). The highest perturbation of ligand activity was observed with N(SO_2_(2-nap))dpa complexation with Pt where cytotoxicity against cancerous cells was lowered and a high cytotoxicity was mounted against MCF-10A non-cancerous cells. It can be hypothesized that complexation with Pt would lock and make the ligand conformationally rigid. Hence, its dynamic binding to the proteins or DNA/RNA can be perturbed. However, some ligands may get locked in the correct conformation, rendering them to be more effective once complexed with Pt acting synergistically. It is interesting to note that the N(SO_2_pip)dpa ligand and its Pt complex display a high level of cytotoxicity against MCF-7 cells. Comparing the IC_50_ values at 24 h against MCF-7 breast cancer cell lines, [PtCl_2_(N(SO_2_pip)dpa)], N(SO_2_(2-nap))dpa, N(SO_2_pip)dpa and [PtCl_2_N(SO_2_(1-nap))dpa] compounds exhibit higher toxicity than the reported value for cisplatin (97.86 μM) (https://www.cancerrxgene.org/translation/Drug/1005) which is a widely used anticancer drug. Further investigations are warranted to decipher the cellular mechanisms of cell death due to these compounds.

High antiproliferative activity implies the need of low concentrations of effective doses that may result in lower side-effects in treatments. This assay system does not test for recurring cell proliferation after the drug pressure is lifted. However, at higher doses cells do not appear in the treatments indicating efficient removal of cancer cells. These findings emphasize that the above compounds have the potential to be promising anticancer drug leads.

## 4. Conclusions

Three novel Pt complexes were synthesized in good yield in high purity and characterized by various spectroscopic techniques. It is noteworthy that the splitting of the methylene (–CH_2_) signal to doublets in NMR spectra is not indicative of the denticity of ligands containing the dpa sulfonamide moiety in platinum and rhenium complexes. Structural results of all three complexes established that a rare 8-membered chelate ring is formed in each of the three complexes where the central sulfonamide N is not bound to Pt. X-ray structures are consistent with the optimized structures from DFT. TDDFT calculations indicated that, the key vertical excitations of N(SO_2_(2-nap))dpa and [PtCl_2_(N(SO_2_(2-nap))dpa)] involved the 2-nap-ligand-centered π → π* excitation (^1^LC). In the case of N(SO_2_(2-nap))dpa, strong ^1^LC emission leads to florescence.

We have reported here the first experimental results for anticancer activity for ligands containing the naphthyl dpa sulfonamide derivatives as well as for three novel platinum complexes where all compounds displayed positive anticancer activity. Low IC_50_ values obtained for ligands and complexes confirm that this moiety could indeed be explored towards finding new drugs which possess promising anticancer properties.

## Author contributions

Nadini Thushara: investigation, formal analysis, methodology, data curation, writing original draft. Taniya Darshani: formal analysis, methodology. Sameera R. Samarakoon: investigation, resources, formal analysis, data curation. Inoka C. Perera: investigation, resources, formal analysis. Frank R. Fronczek: investigation, resources, formal analysis, data curation. W. M. C. Sameera: investigation, resources, formal analysis. Theshini Perera: methodology, conceptualization, supervision, writing – review & editing.

## Conflicts of interest

The authors declare no conflict of interest regarding the publication of this paper.

## Supplementary Material

RA-011-D1RA00842K-s001

RA-011-D1RA00842K-s002
