# Supplementary material for: Synthesis, characterization and biological evaluation of dipicolylamine sulfonamide derivatized platinum complexes as potential anticancer agents
Source: RSC Adv. 2021 May 14;11(29):17658–68. doi: 10.1039/d1ra00842k (PMC9033217; doi:10.1039/d1ra00842k)
Supplement: RA-011-D1RA00842K-s001 [file RA-011-D1RA00842K-s001.pdf]

Electronic Supplementary Material (ESI) for RSC Advances.
This journal is © The Royal Society of Chemistry 2021

Synthesis, Characterization and Biological Evaluation of Dipicolylamine Sulfonamide Derivatized Platinum Complexes as Potential Anticancer Agents

Supporting Information

Nadini Thushara[1], Taniya Darshani[1], Sameera R Samarakoon[4], Inoka C Perera[2], Frank R. Fronczek,[3] W M C Sameera[5] and Theshini Perera*[1]

*[1] Department of Chemistry, University of Sri Jayewardenepura, Sri Lanka *theshi@sjp.ac.lk*
[2] Department of Zoology and Environmental Science, University of Colombo, Sri Lanka
[3] Department of Chemistry, Louisiana State University, Baton Rouge, LA, USA
[4]Institute of Biochemistry, Molecular Biology and Biotechnology, University of Colombo, Sri Lanka
[5]Institute of Low Temperature Science, Hokkaido University, N19-W8, Kita ku, Sapporo, Hokkaido 060-0819, Japan.

Section	PAGE
1. Selected bond distances for N(SO_2(2-nap))dpa	2
2. Selected bond angles for N(SO_2(2-nap))dpa	2
3. Experimental excitation (Ex) and emission (Em) wavelengths of compounds	2
4. Emission spectra of Pt complexes in methanol excited at 275 nm	3
**5.** Kohn-Sham frontier molecular orbitals of the optimized ground state structures of [$PtCl_2$(N(SO_2(1-nap))dpa)] and [$PtCl_2$(N(SO_2pip)dpa)] complexes	4
6. TDDFT results for N(SO_2(2-nap))dpa and [$PtCl_2$(N(SO_2(2-nap))dpa)] systems	5
7. IC_{50} graphs of compounds	25
8. IC_{50} graphs of compounds against MCF-10A human normal breast cells	26
9. Cartesian coordinates of the optimized structures	26

1. Selected bond distances for $N(SO_2(2\text{-nap}))dpa$

Table S1. Selected bond distances for $N(SO_2(2\text{-nap}))dpa$

	Bond length (Å)			Bond length (Å)	
	Actual	Calculated		Actual	Calculated
S1-O1	1.4350 (6)	1.4324	S1-C13	1.7692 (6)	1.7688
S1-O2	1.4352 (6)	1.4333	C13-C22	1.4191 (9)	1.4140
S1-N2	1.6277 (6)	1.6314	N2-C7	1.4712 (9)	1.4584
S1-C13	1.7692 (6)	1.7653	N2-C6	1.4752 (9)	1.4613
N1-C5	1.3359 (10)	1.3306	N3-C8	1.3401 (10)	1.3313
N1-C1	1.3462 (11)	1.3317	N3-C12	1.3412 (11)	1.3303

2. Selected bond angles for $N(SO_2(2\text{-nap}))dpa$

Table S2. Selected bond angles for $N(SO_2(2\text{-nap}))dpa$

	Bond angle (deg)			Bond angle (deg)	
	Actual	Calculated		Actual	Calculated
O2-S1-O1	119.64 (4)	119.83	C5-N1-C1	117.09 (7)	117.98
O2-S1-N2	106.94 (3)	107.10	C7-N2-C6	117.13 (6)	116.42
O1-S1-N2	106.83 (3)	106.95	C7-N2-S1	115.64 (5)	117.96
O2-S1-C13	107.29 (3)	108.11	C6-N2-S1	119.61 (5)	119.67
O1-S1-C13	107.43 (3)	107.99	C8-N3-C12	117.61 (7)	118.16
N2-S1-C13	108.30 (3)	106.05	N1-C1-C2	123.93 (8)	123.38

3. Experimental emission (Em) wavelengths of compounds

Table S3. Experimental emission (Em) wavelengths of $N(SO_2(2\text{-nap}))dpa$, $[PtCl_2(N(SO_2(2\text{-nap}))dpa)]$, $N(SO_2(1\text{-nap}))dpa$, $[PtCl_2(N(SO_2(1\text{-nap}))dpa)]$ and $[PtCl_2(NSO_2pipdpa)]$ in methanol.

Test sample	Em (nm)
$N(SO_2(2\text{-nap}))dpa$	343
$[PtCl_2(N(SO_2(2\text{-nap}))dpa)]$	612
$N(SO_2(1\text{-nap}))dpa$	342
$[PtCl_2(N(SO_2(1\text{-nap}))dpa)]$	615
$[PtCl_2(N(SO_2pip)dpa)]$	611

4. Emission spectra of Pt complexes in methanol excited at 275 nm

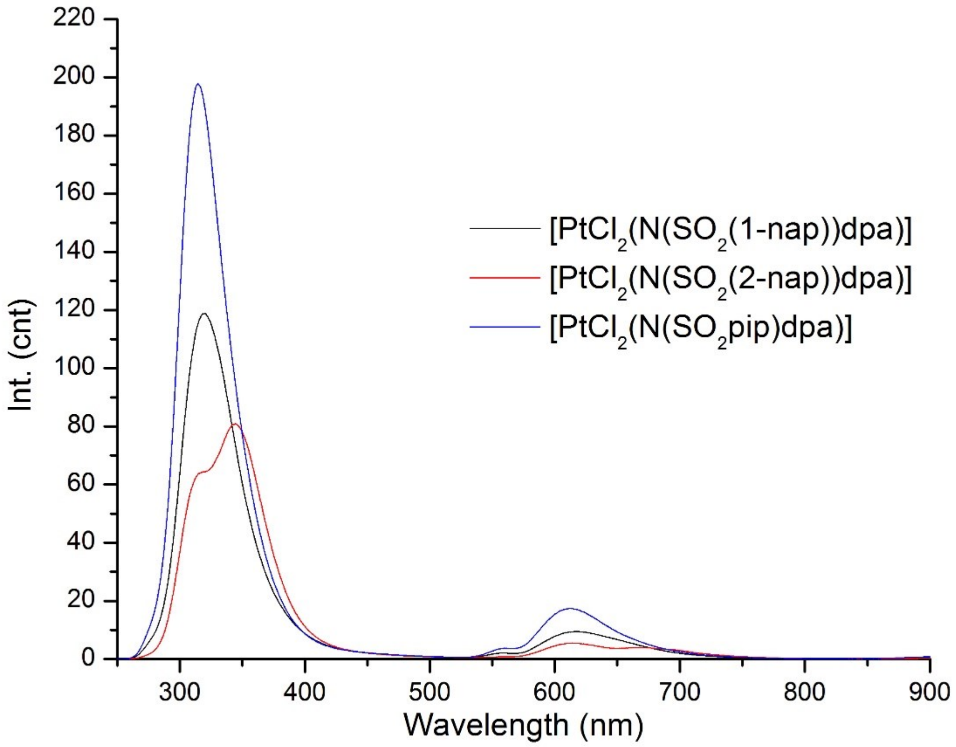

Figure S1: Emission spectra of Pt complexes in methanol excited at 275nm

5. Kohn-Sham frontier molecular orbitals of the optimized ground state structures of [$PtCl_2$(N(SO_2(1-nap)dpa))] and [$PtCl_2$(N(SO_2pip)dpa))] complexes

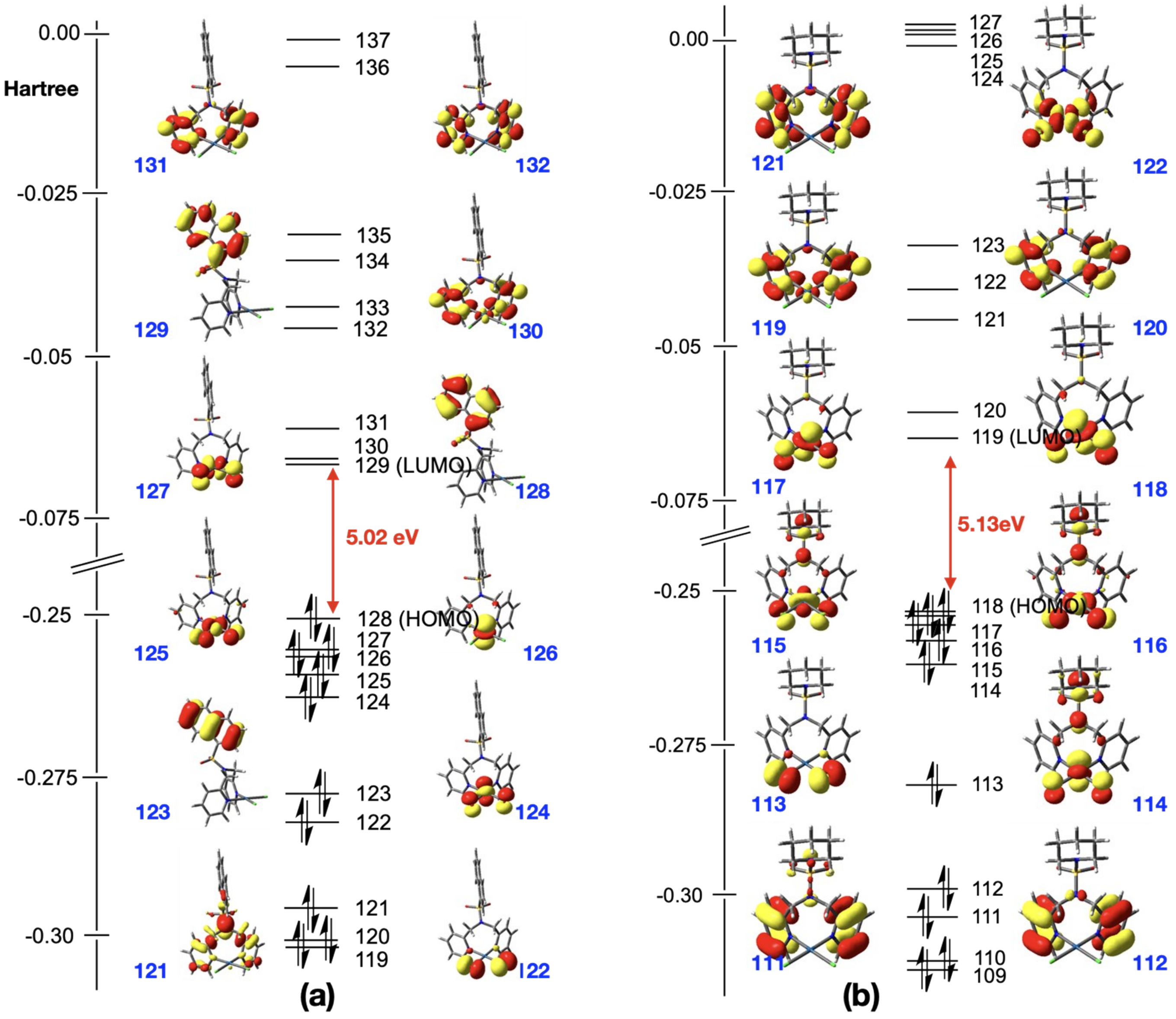

Figure S2. Kohn-Sham frontier molecular orbitals of the optimized structures of (a) [$PtCl_2$(N(SO_2((1-nap))dpa)] and (b) [$PtCl_2$(N(SO_2pip)dpa)].

6. TDDFT results for N(SO_2(2-nap))dpa and [$PtCl_2$(N(SO_2(2-nap))dpa)] systems

N(SO_2(2-nap))dpa system

Excited State 1: Singlet-?Sym 4.2868 eV 289.23 nm f=0.0651 <S**2>=0.000

101 ->103 0.16166

102 ->103 0.63276

102 ->104 -0.13264

102 ->105 0.11826

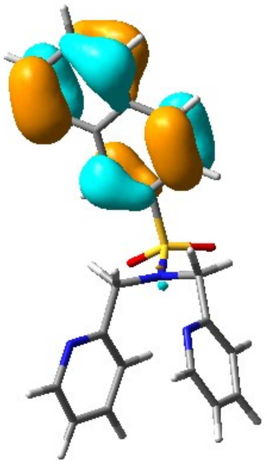

HOTO

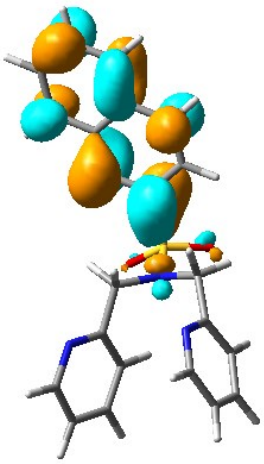

LUTO

This state for optimization and/or second-order correction.

Total Energy, E(TD-HF/TD-DFT) = -1561.53864624

Copying the excited state density for this state as the 1-particle RhoCI density.

Excited state symmetry could not be determined.

Excited State 2: Singlet-?Sym 4.4607 eV 277.95 nm f=0.0170 <S**2>=0.000

97 ->103 -0.10183

99 ->103 0.13310

100 ->103 0.16912

101 ->103 0.37935

102 ->103 -0.26780

102 ->104 -0.37215

102 ->105 0.25788

Excited state symmetry could not be determined.

Excited State 3: Singlet-?Sym 4.9643 eV 249.75 nm f=0.1671 <S**2>=0.000

98 ->103 -0.16725

98 ->104 0.22833

98 ->105 0.26700

98 ->106 0.13534

98 ->107 -0.18253

99 ->103 0.11099

99 ->104 -0.14775

99 ->105 -0.11032

99 ->106 0.12858

101 ->103 -0.24534

101 ->104 0.18181

101 ->105 0.14153

102 ->104 -0.25931

Excited state symmetry could not be determined.

Excited State 4: Singlet-?Sym 5.0036 eV 247.79 nm f=0.0083 <S**2>=0.000

98 ->103 0.11255

98 ->104 -0.24101

98 ->105 -0.27832

99 ->104 -0.26691

99 ->105 -0.20012

99 ->106 0.28264

99 ->107 -0.21859

101 ->104 0.11553

101 ->106 -0.15157

101 ->107 0.11551

Excited state symmetry could not be determined.

Excited State 5: Singlet-?Sym 5.0776 eV 244.18 nm f=0.2415 <S**2>=0.000

96 ->103 0.10542

99 ->104 -0.12325

99 ->105 -0.10136

99 ->106 0.11893

100 ->103 -0.38470

100 ->104 0.10280

101 ->103 0.43933

102 ->104 0.13514

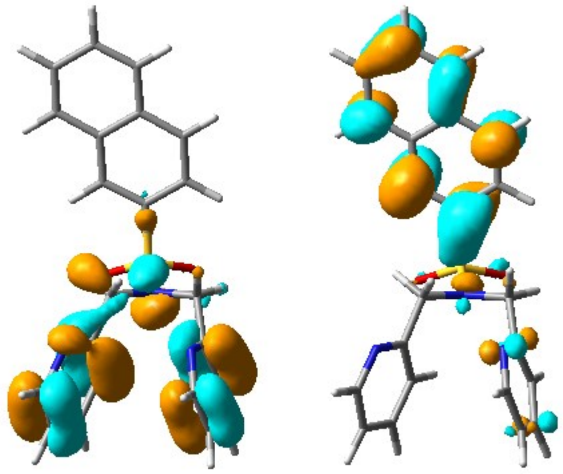

HOTO **LUTO**

Excited state symmetry could not be determined.

Excited State 6: Singlet-?Sym 5.1089 eV 242.68 nm f=0.2962 <S**2>=0.000

96 ->103 0.12567

97 ->103 0.11427

98 ->104 0.12184

98 ->105 0.17995

98 ->106 0.12539

99 ->103 -0.10048

100 ->103 0.49784

101 ->103 0.11447

102 ->104 0.25705

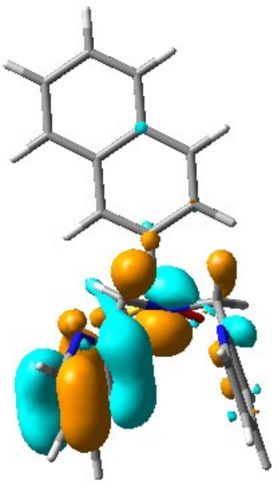

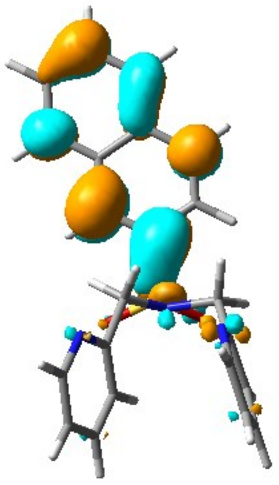

HOTO **LUTO**

Excited state symmetry could not be determined.

Excited State 7: Singlet-?Sym 5.2126 eV 237.86 nm f=0.0031 <S**2>=0.000

98 ->103 -0.13284

99 ->103 0.18435

100 ->103 -0.14726

102 ->104 0.34330

102 ->105 0.50265

Excited state symmetry could not be determined.

Excited State 8: Singlet-?Sym 5.2607 eV 235.68 nm f=0.0044 <S**2>=0.000

98 ->104 -0.10312

98 ->105 0.11165

98 ->106 0.35890

98 ->107 0.37067

98 ->108 -0.17284

99 ->103 0.13565

100 ->105 0.12211

100 ->107 0.10377

101 ->104 -0.10232

101 ->106 0.12306

101 ->107 0.11776

102 ->106 -0.16523

102 ->107 -0.13352

Excited state symmetry could not be determined.

Excited State 9: Singlet-?Sym 5.3032 eV 233.79 nm f=0.0306 <S**2>=0.000

95 ->106 -0.10350

100 ->103 0.15625

100 ->104 0.37669

100 ->105 0.32562

100 ->106 -0.11139

101 ->104 -0.21416

101 ->105 -0.22494

102 ->106 0.11737

Excited state symmetry could not be determined.

Excited State 10: Singlet-?Sym 5.3772 eV 230.58 nm f=0.1415 <S**2>=0.000

98 ->106 0.17101

98 ->107 0.16086

99 ->103 -0.30685

99 ->105 0.10291

99 ->106 0.14851

99 ->107 0.11380

99 ->108 0.23771

101 ->104 0.13725

101 ->108 -0.12633

102 ->105 0.22599

102 ->106 0.31474

Excited state symmetry could not be determined.

Excited State 11: Singlet-?Sym 5.4255 eV 228.52 nm f=0.1441 <S**2>=0.000

97 ->103 0.11312

98 ->103 0.10344

99 ->103 0.41816

99 ->105 0.10043

99 ->106 0.13598

99 ->107 0.17871

99 ->108 0.25353

100 ->104 -0.11156

100 ->107 -0.10820

101 ->106 -0.15049

101 ->108 -0.11848

102 ->105 -0.12361

102 ->107 0.15587

Excited state symmetry could not be determined.

Excited State 12: Singlet-?Sym 5.4390 eV 227.95 nm f=0.0082 <S**2>=0.000

99 ->103 0.20135

99 ->106 -0.16286

99 ->108 -0.22272

102 ->106 0.52072

102 ->107 -0.12024

Excited state symmetry could not be determined.

Excited State 13: Singlet-?Sym 5.4691 eV 226.70 nm f=0.0469 <S**2>=0.000

98 ->103 0.46091

100 ->104 0.16426

101 ->104 0.17124

101 ->107 -0.10530

102 ->105 0.13361

102 ->107 0.35507

Excited state symmetry could not be determined.

Excited State 14: Singlet-?Sym 5.4963 eV 225.58 nm f=0.0258 <S**2>=0.000

97 ->105 -0.10049

98 ->103 -0.20123

99 ->103 -0.16593

99 ->108 -0.12099

100 ->106 0.19496

100 ->107 -0.10315

101 ->104 -0.16319

102 ->107 0.46401

Excited state symmetry could not be determined.

Excited State 15: Singlet-?Sym 5.5432 eV 223.67 nm f=0.1390 <S**2>=0.000

94 ->107 0.12480

95 ->108 0.13849

97 ->104 -0.11111

97 ->105 -0.13658

98 ->103 0.21419

99 ->103 -0.12563

100 ->106 0.34110

100 ->107 -0.10003

101 ->104 0.19453

101 ->105 -0.19620

101 ->106 -0.15902

102 ->107 -0.22615

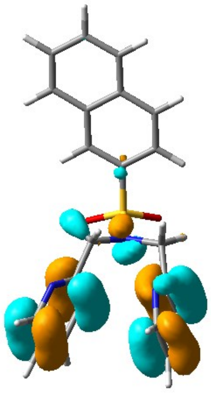

HOTO

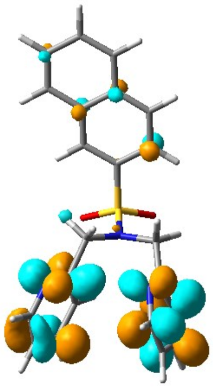

LUTO

Excited state symmetry could not be determined.

Excited State 16: Singlet-?Sym 5.5691 eV 222.63 nm f=0.0291 <S**2>=0.000

97 ->103 0.51715

98 ->103 -0.25483

98 ->107 0.12523

101 ->104 0.28321

Excited state symmetry could not be determined.

Excited State 17: Singlet-?Sym 5.6325 eV 220.12 nm f=0.3422 <S**2>=0.000

97 ->103 0.26505

98 ->103 0.15067

98 ->107 -0.10013

99 ->106 0.10228

99 ->107 -0.10401

100 ->104 -0.12267

100 ->105 -0.25727

100 ->106 -0.17153

100 ->107 0.14791

101 ->104 -0.25454

101 ->105 -0.20783

101 ->106 0.10792

102 ->104 -0.17950

102 ->106 -0.12352

Excited state symmetry could not be determined.

Excited State 18: Singlet-?Sym 5.7063 eV 217.28 nm f=0.1358 <S**2>=0.000

96 ->103 -0.18879

96 ->105 0.11145

97 ->103 0.24773

100 ->105 0.11517

100 ->107 -0.11027

101 ->104 -0.22353

101 ->105 0.46062

102 ->105 0.12804

Excited state symmetry could not be determined.

Excited State 19: Singlet-?Sym 5.7256 eV 216.54 nm f=0.0154 <S**2>=0.000

100 ->104 -0.36421

100 ->105 0.32171

100 ->107 0.40300

101 ->107 -0.23555

Excited state symmetry could not be determined.

Excited State 20: Singlet-?Sym 5.7737 eV 214.74 nm f=0.1385 <S**2>=0.000

93 ->103 -0.23409

96 ->103 0.39177

97 ->104 0.14998

99 ->105 0.10048

101 ->106 -0.13230

102 ->108 -0.13241

102 ->109 0.38003

Excited state symmetry could not be determined.

Excited State 21: Singlet-?Sym 5.7927 eV 214.03 nm f=0.0061 <S**2>=0.000

96 ->103 0.22175

97 ->106 0.10403

99 ->104 -0.23466

99 ->105 -0.12896

100 ->106 0.17102

101 ->106 0.41577

101 ->107 -0.24871

102 ->106 0.10264

102 ->107 -0.10198

Excited state symmetry could not be determined.

Excited State 22: Singlet-?Sym 5.8145 eV 213.23 nm f=0.0210 <S**2>=0.000

93 ->103 0.12667

97 ->103 0.10945

97 ->104 0.20418

97 ->105 0.25967

97 ->106 0.11386

99 ->105 0.10717

99 ->106 0.11966

99 ->107 -0.11588

100 ->105 0.20934

100 ->106 0.16295

101 ->106 0.17584

101 ->107 0.21508

102 ->108 0.18885

102 ->109 -0.16894

Excited state symmetry could not be determined.

Excited State 23: Singlet-?Sym 5.8324 eV 212.58 nm f=0.0160 <S**2>=0.000

93 ->103 0.10303

96 ->103 0.12172

100 ->104 0.24820

100 ->105 -0.24250

100 ->106 0.30841

100 ->107 0.35195

101 ->104 -0.11678

101 ->105 0.12471

101 ->106 -0.14329

101 ->107 -0.10376

102 ->108 0.18408

102 ->109 -0.11908

Excited state symmetry could not be determined.

Excited State 24: Singlet-?Sym 5.8608 eV 211.55 nm f=0.0223 <S**2>=0.000

96 ->103 0.19093

97 ->104 -0.17662

97 ->105 -0.16147

97 ->107 0.10984

98 ->106 -0.14097

99 ->108 0.12392

100 ->106 -0.12193

100 ->107 0.10901

101 ->105 0.13757

101 ->106 0.14126

101 ->107 0.30626

101 ->108 -0.10301

102 ->108 0.27833

Excited state symmetry could not be determined.

Excited State 25: Singlet-?Sym 5.8898 eV 210.51 nm f=0.1247 <S**2>=0.000

96 ->103 0.22552

99 ->105 -0.10836

99 ->108 -0.10793

100 ->104 -0.10129

100 ->106 -0.17044

100 ->107 -0.21447

101 ->106 -0.19519

101 ->107 -0.25541

102 ->108 0.35906

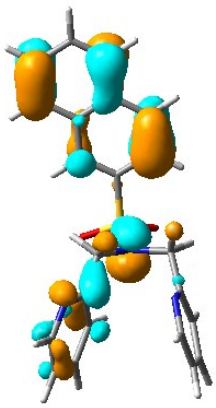

HOTO

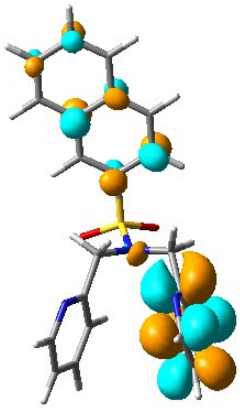

LUTO

Excited state symmetry could not be determined.

Excited State 26: Singlet-?Sym 5.9254 eV 209.24 nm f=0.0849 <S**2>=0.000

93 ->103 -0.24565

96 ->103 -0.27757

97 ->104 0.10287

98 ->104 0.10845

99 ->104 -0.15420

102 ->108 0.38867

102 ->109 0.29626

Excited state symmetry could not be determined.

Excited State 27: Singlet-?Sym 5.9708 eV 207.65 nm f=0.0906 <S**2>=0.000

93 ->103 -0.12878

97 ->104 -0.14813

98 ->104 -0.31343

98 ->106 0.22397

98 ->107 -0.28428

99 ->104 0.31667

99 ->106 0.11622

99 ->107 -0.12309

101 ->104 0.11537

102 ->108 0.10084

102 ->109 0.13354

Excited state symmetry could not be determined.

Excited State 28: Singlet-?Sym 5.9857 eV 207.14 nm f=0.0108 <S**2>=0.000

97 ->104 0.27469

97 ->106 -0.26678

97 ->107 0.19965

98 ->104 -0.16654

98 ->106 0.17415

98 ->107 -0.21082

99 ->104 -0.18311

99 ->105 -0.10650

99 ->106 -0.22837

99 ->107 0.19096

101 ->107 0.12036

Excited state symmetry could not be determined.

Excited State 29: Singlet-?Sym 6.0560 eV 204.73 nm f=0.0728 <S**2>=0.000

96 ->104 0.16803

97 ->105 -0.10400

98 ->106 0.12610

99 ->104 -0.27945

99 ->105 0.50735

99 ->108 -0.14096

101 ->105 0.10374

101 ->108 0.11374

Excited state symmetry could not be determined.

Excited State 30: Singlet-?Sym 6.0776 eV 204.00 nm f=0.0062 <S**2>=0.000

95 ->103 0.66679

[$PtCl_2$(N(SO_2(2-nap))dpa)] system

Excitation energies and oscillator strengths:

Excited state symmetry could not be determined.

Excited State 1: Singlet-?Sym 3.6564 eV 339.08 nm f=0.0002 <S**2>=0.000

126 -> 133 0.64213

126 -> 134 0.14491

127 -> 133 -0.17437

128 -> 133 0.13091

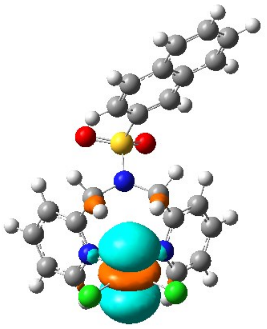

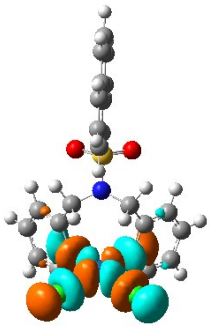

HOTO **LUTO**

Excited State 2: Singlet-?Sym 3.7971 eV 326.52 nm f=0.0007 <S**2>=0.000

124 -> 133 0.15356

125 -> 133 0.64762

125 -> 134 0.14541

Excited State 3: Singlet-?Sym 3.8400 eV 322.87 nm f=0.0003 <S**2>=0.000

115 -> 133 0.12577

126 -> 133 0.16908

127 -> 133 0.64223

127 -> 134 0.14403

Excited state symmetry could not be determined.

Excited State 4: Singlet-?Sym 3.9452 eV 314.26 nm f=0.0006 <S**2>=0.000

114 -> 133 -0.11033

124 -> 133 0.60912

124 -> 134 0.13579

125 -> 133 -0.13554

127 -> 129 -0.16861

127 -> 130 0.14689

Excited State 5: Singlet-?Sym 3.9979 eV 310.12 nm f=0.0003 <S**2>=0.000

126 -> 129 0.49463

126 -> 130 -0.43537

127 -> 129 -0.13272

127 -> 130 0.11942

128 -> 129 0.12138

128 -> 130 -0.10163

Excited State 6: Singlet-?Sym 4.1498 eV 298.77 nm f=0.0002 <S**2>=0.000

126 -> 129 0.14560

126 -> 130 0.11171

126 -> 131 0.54325

127 -> 129 0.22058

127 -> 130 -0.25888

127 -> 131 -0.11840

128 -> 131 0.13382

Excited State 7: Singlet-?Sym 4.1561 eV 298.32 nm f=0.0004 <S**2>=0.000

124 -> 133 0.18358

126 -> 130 -0.19505

126 -> 131 -0.30113

127 -> 129 0.42928

127 -> 130 -0.32986

127 -> 131 0.13647

Excited State 8: Singlet-?Sym 4.2545 eV 291.42 nm f=0.0094 <S**2>=0.000

124 -> 129 0.39745

124 -> 130 -0.36024

125 -> 129 -0.14635

125 -> 130 0.13510

127 -> 131 -0.30099

128 -> 129 -0.20146

Excited State 9: Singlet-?Sym 4.2665 eV 290.60 nm f=0.0472 <S**2>=0.000

123 -> 129 0.11884

123 -> 130 0.12685

123 -> 132 0.11165

124 -> 129 0.12759

127 -> 131 -0.12942

128 -> 129 0.44555

128 -> 130 0.36584

128 -> 131 -0.11746

128 -> 134 0.15196

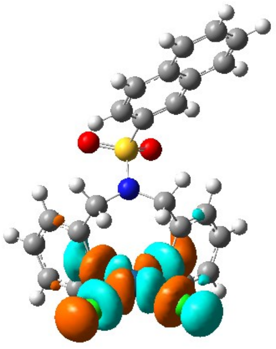

HOTO

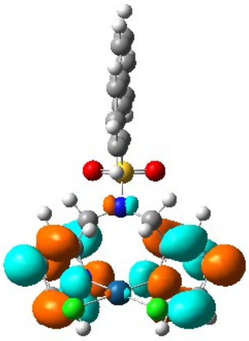

LUTO

Excited State 10: Singlet-?Sym 4.2821 eV 289.54 nm f=0.0005 <S**2>=0.000

124 -> 129 0.27186

124 -> 130 -0.22822

126 -> 131 0.14721

127 -> 130 0.18073

127 -> 131 0.54097

Excited State 11: Singlet-?Sym 4.3982 eV 281.89 nm f=0.0139 <S**2>=0.000

124 -> 130 0.17764

124 -> 131 0.50464

125 -> 129 -0.11139

125 -> 131 -0.39154

128 -> 131 -0.12723

Excited State 12: Singlet-?Sym 4.4081 eV 281.27 nm f=0.0562 <S**2>=0.000

124 -> 129 0.17263

124 -> 130 -0.10635

125 -> 129 0.49304

125 -> 130 -0.43023

Excited State 13: Singlet-?Sym 4.4514 eV 278.53 nm f=0.0536 <S**2>=0.000

124 -> 130 0.12096

124 -> 131 0.40352

125 -> 130 0.16800

125 -> 131 0.51053

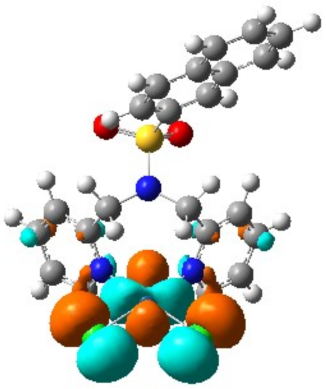

HOTO

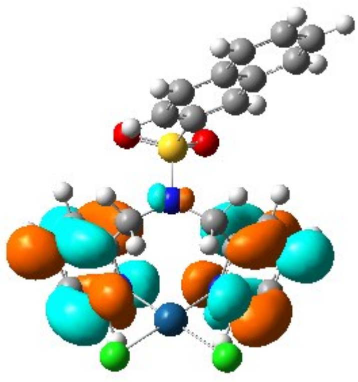

LUTO

Excited State 14: Singlet-?Sym 4.4760 eV 277.00 nm f=0.0291 <S**2>=0.000

123 -> 129 0.32742

123 -> 130 0.24047

128 -> 129 -0.26463

128 -> 130 -0.10373

128 -> 132 -0.28380

128 -> 134 0.31203

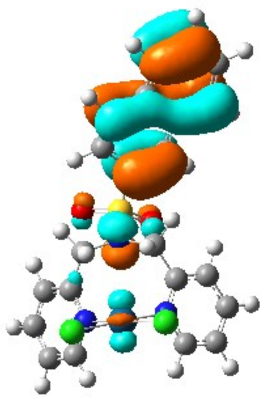

HOTO

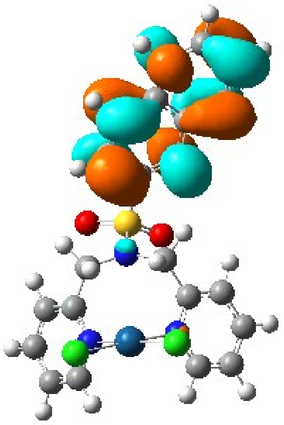

LUTO

Excited State 15: Singlet-?Sym 4.5467 eV 272.69 nm f=0.0021 <S**2>=0.000

126 -> 129 0.14316

128 -> 129 -0.34901

128 -> 130 0.55483

Excited State 16: Singlet-?Sym 4.5806 eV 270.68 nm f=0.0028 <S**2>=0.000

126 -> 129 0.35480

126 -> 130 0.39542

126 -> 131 -0.17077

126 -> 132 -0.28295

126 -> 134 -0.16242

127 -> 129 -0.10737

127 -> 130 -0.11712

128 -> 132 -0.14148

Excited State 17: Singlet-?Sym 4.6428 eV 267.05 nm f=0.0001 <S**2>=0.000

126 -> 129 0.11558

126 -> 130 0.12562

127 -> 129 0.40305

127 -> 130 0.43365

127 -> 131 -0.20934

127 -> 132 -0.22334

127 -> 134 -0.10870

Excited State 18: Singlet-?Sym 4.6563 eV 266.27 nm f=0.0025 <S**2>=0.000

123 -> 131 -0.10342

126 -> 131 -0.15117

128 -> 129 0.16085

128 -> 131 0.64099

Excited State 19: Singlet-?Sym 4.7503 eV 261.00 nm f=0.0123 <S**2>=0.000

125 -> 129 0.39151

125 -> 130 0.43218

125 -> 131 -0.21108

125 -> 132 -0.25900

125 -> 134 -0.13206

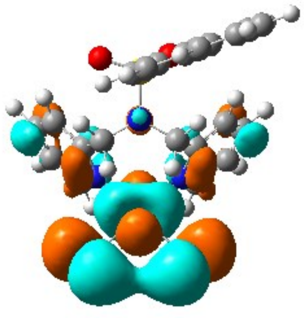

HOTO

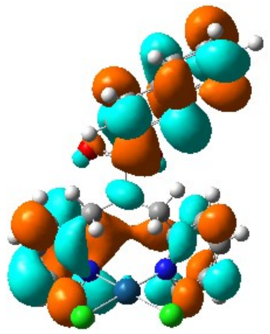

LUTO

Excited State 20: Singlet-?Sym 4.7970 eV 258.46 nm f=0.0067 <S**2>=0.000

124 -> 129 -0.24169

124 -> 130 -0.24892

124 -> 131 0.12264

124 -> 132 0.15029

126 -> 129	0.17427
126 -> 130	0.18947
126 -> 132	0.38417
126 -> 134	0.23626
127 -> 132	-0.12200
128 -> 134	0.10105

7. IC_{50} graphs of compounds against MCF-7 human breast cancer cells

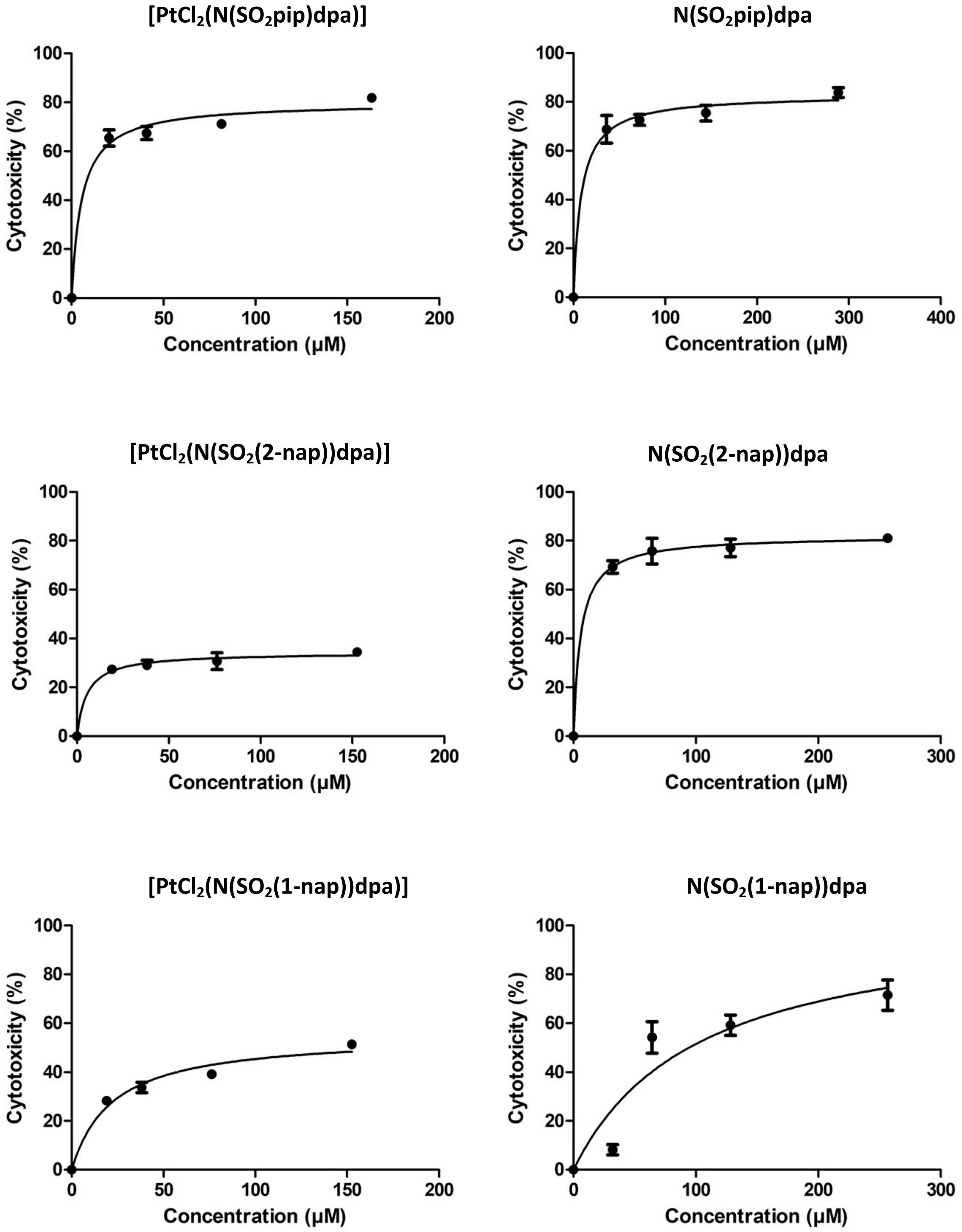

Fig S3. IC_{50} graphs of compounds

8. IC_{50} graphs of compounds against MCF-10A human normal breast cells

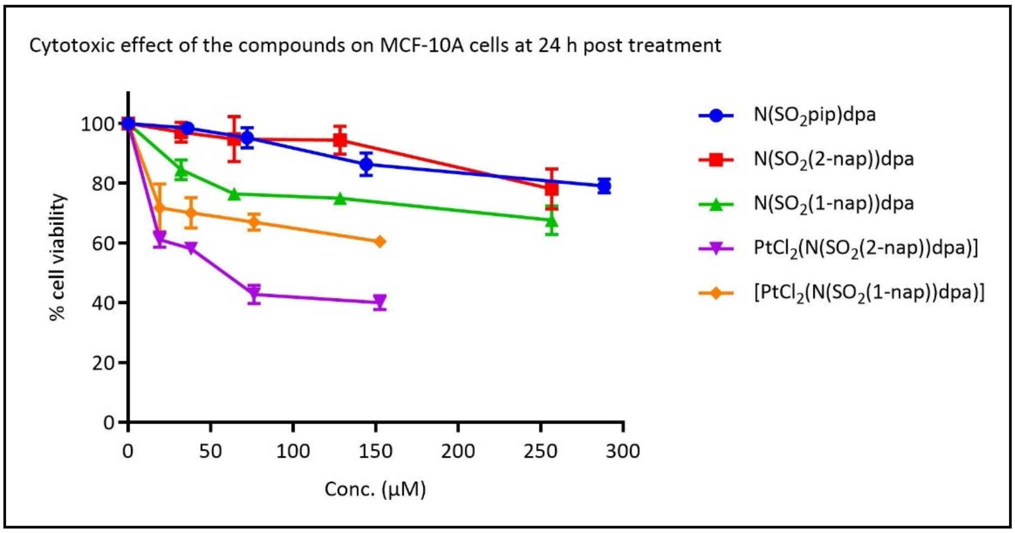

9. Cartesian coordinates of the optimized structures

N(SO_2(2-nap))dpa system

S 4.54194000 1.71050500 5.13762800
O 4.32082900 0.56066200 4.30771000
O 4.90949300 1.54382500 6.51548200
N 0.69181200 4.46903600 5.99196600
N 3.15920000 2.57659000 5.11818000
N 0.34574800 2.62471000 2.79941700
C -0.59183600 4.43864300 6.34261600
H -1.24542700 5.14048900 5.83295800
C -1.10193600 3.57542200 7.29848900
H -2.15677600 3.59211600 7.54214400
C -0.22820400 2.70485700 7.93206200
H -0.58339500 2.01913000 8.69227500
C 1.10677100 2.72582100 7.57164600
H 1.82254000 2.05441900 8.03112600
C 1.52200500 3.61810000 6.59007800
C 2.95041100 3.62256900 6.11817900
H 3.18378400 4.60332400 5.69244000
H 3.61816700 3.42393200 6.95401800
C 2.49848100 2.76481600 3.83682400
H 3.13092400 2.36542300 3.04002100
H 2.38351800 3.83412600 3.64323000
C 1.14212800 2.11447700 3.73642900
C 0.76705200 1.04810000 4.54143600
H 1.45152900 0.66617800 5.28667100
C -0.49481200 0.50333700 4.37601200
H -0.81973600 -0.32374000 4.99663700
C -1.33270600 1.03290800 3.40764300
H -2.32908000 0.64142300 3.24567400
C -0.86065300 2.08788800 2.64292700
H -1.48554100 2.52789900 1.87082600
C 5.76352000 2.70958400 4.36189600
C 6.09688200 2.47660400 3.05487300

H 5.65573500 1.64523200 2.51849200
C 7.02985500 3.31268600 2.40589000
C 7.40190700 3.10396100 1.05952900
H 6.95702400 2.27801800 0.51567500
C 8.30697200 3.93215700 0.45400600
H 8.58714200 3.76743300 -0.57968800
C 8.88020800 5.00292000 1.16800700
H 9.59602500 5.65052000 0.67537700
C 8.53990500 5.22824100 2.47463300
H 8.98137900 6.05125300 3.02572100
C 7.60835500 4.39304400 3.12763600
C 7.23834800 4.59511500 4.47784400
H 7.68966400 5.41361100 5.02732800
C 6.33561900 3.77527600 5.09060500
H 6.06891200 3.92487300 6.12897000

[$PtCl_2$(N(SO_2(1-nap))dpa)] complex

Pt 6.77147200 4.57946800 10.39376400
Cl 5.91861200 5.12465400 8.30810400
Cl 4.80423500 5.18225800 11.46251500
N 8.49844600 3.99009300 9.49741000
N 9.76957300 6.22642300 11.41342700
N 7.54559500 4.05072000 12.19891600
C 8.53625600 2.75082100 8.99031600
H 7.63113200 2.16859900 9.09301700
C 9.65596800 2.24459000 8.36582200
H 9.63497400 1.23785700 7.97114600
C 10.77696400 3.04913500 8.25580200
H 11.67319500 2.68806800 7.76689500
C 10.73146200 4.32736900 8.78100300
H 11.58632200 4.98716300 8.72675300
C 9.57991600 4.78048300 9.40374100
C 9.51633000 6.16874100 9.97562600
H 10.25512100 6.78346900 9.46310100
H 8.53123900 6.59914300 9.78379800
C 8.66819300 6.22272400 12.37338400
H 7.78674200 6.64104200 11.88350400
H 8.92641400 6.87230800 13.20882700
C 8.33975200 4.86091100 12.91736700
C 8.83618600 4.45310500 14.14473900
H 9.47677000 5.12764000 14.69579600
C 8.52131300 3.19984200 14.63661400
H 8.90494300 2.87400000 15.59541700
C 7.70628700 2.37443900 13.88138400
H 7.42564700 1.38581900 14.21854300
C 7.23633000 2.83565800 12.67037400
H 6.58737500 2.23500800 12.04824400
S 11.25819000 6.61825500 11.93274100
O 12.19343500 6.13224200 10.95704600
O 11.36715200 6.17313000 13.29392600
C 11.34540600 8.38519100 11.93280400
C 10.26047500 9.11469600 11.52640500
H 9.35253500 8.62068700 11.20771300
C 10.30933300 10.51960300 11.51756300
H 9.44057700 11.07819600 11.19276800
C 11.44466900 11.16413600 11.91630500
H 11.49209700 12.24743000 11.91303200
C 12.58162700 10.44069000 12.34134500
C 13.75462700 11.11029500 12.75337600
H 13.76383800 12.19450100 12.73706200
C 14.85399500 10.40893900 13.16382900

H 15.74959800 10.93134500 13.47829200
C 14.82317000 9.00304900 13.17825700
H 15.69694200 8.45122600 13.50419300
C 13.70232600 8.32195300 12.78562900
H 13.70439300 7.24055100 12.80652200
C 12.54985100 9.01728100 12.35638100

[$PtCl_2(N(SO_2(2\text{-}nap))dpa)$] complex

Pt 18.24248900 4.86333400 7.73597600
Cl 18.93409100 5.44651700 9.86989100
Cl 20.29192600 5.43770700 6.81395300
S 13.87607400 6.71030300 5.77574300
N 16.45112000 4.30614700 8.51993000
N 15.33831800 6.44948200 6.45380900
N 17.62128700 4.29729600 5.88310800
C 16.36450400 3.08196900 9.05606700
H 17.26435100 2.48338700 9.02138800
C 15.20270900 2.60989300 9.62862600
H 15.18424900 1.61389500 10.04976800
C 14.09103900 3.43443000 9.65413200
H 13.16301100 3.10020900 10.10107200
C 14.18685700 4.69658800 9.09741700
H 13.34109300 5.37040100 9.08331800
C 15.37920800 5.11476700 8.52943000
C 15.49364100 6.47590800 7.90263300
H 14.72851100 7.11825200 8.33415400
H 16.47059200 6.90512900 8.13067500
C 16.49590500 6.45197300 5.56891200
H 17.32998000 6.91274200 6.10100800
H 16.27725200 7.08230900 4.70426000
C 16.90019500 5.09172800 5.07545500
C 16.54522200 4.66687700 3.80562700
H 15.96661100 5.32943900 3.17686900
C 16.91890500 3.41083400 3.36508700
H 16.64480900 3.07235000 2.37365000
C 17.64853300 2.59838600 4.21556500
H 17.96773300 1.60704000 3.92382000
C 17.98421100 3.07821100 5.46340100
H 18.56822000 2.49118500 6.15847600
O 12.91977200 6.64967100 6.84495600
O 13.74534400 5.83883600 4.64364500
C 13.93973800 8.35719100 5.16702200
C 14.02165300 8.57699000 3.81920800
H 14.01938300 7.74162100 3.12978900
C 14.09813300 9.89655900 3.32460900
C 14.18004200 10.16545000 1.94105000
H 14.18457800 9.33378300 1.24523200
C 14.25184200 11.45502200 1.48916100
H 14.31426000 11.65552600 0.42616700
C 14.24453300 12.52888500 2.40045300
H 14.30139300 13.54501000 2.02809900
C 14.16591000 12.29769400 3.74776700
H 14.15950700 13.12413500 4.44971500
C 14.09111700 10.97983800 4.24567300
C 14.00550400 10.70571900 5.63176500
H 13.99478100 11.53539700 6.32955200
C 13.93120600 9.42404300 6.09213200
H 13.85346700 9.22235700 7.15293300

[$PtCl_2(N(SO_2pip)dpa))$] complex

Pt 4.93334600 4.69948400 6.25613400
Cl 2.81452500 4.13415200 5.50621400
Cl 5.90023400 4.20958000 4.20640600
S 6.76864300 2.39638100 10.55346700
O 5.77724600 2.71379900 11.53687000
O 8.09458600 2.92896100 10.64750100
N 4.13273600 5.18897000 8.05827400
N 6.16025400 2.91907900 9.12322700
N 6.75641700 5.25164100 6.96782600
N 6.92325500 0.77472200 10.55437400
C 3.61057900 6.41546800 8.18840700
H 3.63518200 7.03906000 7.30535700
C 3.06462400 6.85997300 9.37344700
H 2.65284000 7.85858800 9.42768000
C 3.05398100 6.00525700 10.46261400
H 2.62919000 6.31811600 11.40833700
C 3.59248700 4.73981300 10.32019500
H 3.61154800 4.04081700 11.14502500
C 4.13239800 4.34814000 9.10540100
C 4.71571100 2.97279500 8.93411300
H 4.23626900 2.31364300 9.65459100
H 4.48304700 2.60137800 7.93423400
C 7.05922800 3.03589400 7.98116100
H 6.55393600 2.64142000 7.09742700
H 7.94060700 2.42254200 8.15779100
C 7.52661300 4.43930600 7.70961800
C 8.74248800 4.88325000 8.20522400
H 9.33609600 4.20626200 8.80391300
C 9.16901900 6.17182500 7.94302000
H 10.11716500 6.52581400 8.32833500
C 8.36300000 6.99602300 7.17648500
H 8.64891900 8.01106600 6.93621700
C 7.16646500 6.49978600 6.70564700
H 6.50487600 7.09954500 6.09616500
C 8.01702200 0.18000400 9.78182300
H 8.88495300 0.83257100 9.85474500
H 7.72881200 0.09566300 8.72411600
C 8.33168200 -1.19911800 10.33055900
H 8.71300500 -1.09448200 11.35136400
H 9.12871600 -1.63992800 9.72671200
C 7.09469700 -2.08252500 10.32600700
H 7.31711900 -3.05464000 10.77220900
H 6.78745400 -2.27022100 9.29022200
C 5.95858600 -1.40308500 11.07288700
H 5.03889500 -1.98895200 11.00191400
H 6.21020000 -1.31581100 12.13468200
C 5.69054600 -0.01545900 10.51993500
H 5.32386700 -0.09096500 9.48604400
H 4.93684600 0.49382000 11.11829100
